# Identification of Diagnostic Markers Correlated With HIV^+^ Immune Non-response Based on Bioinformatics Analysis

**DOI:** 10.3389/fmolb.2021.809085

**Published:** 2021-12-22

**Authors:** Ruojing Bai, Zhen Li, Yuying Hou, Shiyun Lv, Ran Wang, Wei Hua, Hao Wu, Lili Dai

**Affiliations:** ^1^ Beijing Key Laboratory for HIV/AIDS Research, Center for Infectious Diseases, Beijing Youan Hospital, Capital Medical University, Beijing, China; ^2^ Institute of Neurology, Tianjin Third Central Hospital Affiliated to Nankai University, Tianjin, China; ^3^ Travel Clinic, Center for Infectious Diseases, Beijing Youan Hospital, Capital Medical University, Beijing, China

**Keywords:** INRs, IRs, bioinformatic gene analysis, gene expression omnibus, diagnostic markers

## Abstract

**Background:** HIV-infected immunological non-responders (INRs) are characterized by their inability to reconstitute CD4^+^ T cell pools after antiretroviral therapy. The risk of non-AIDS-related diseases in INRs is increased, and the outcome and prognosis of INRs are inferior to that of immunological responders (IRs). However, few markers can be used to define INRs precisely. In this study, we aim to identify further potential diagnostic markers associated with INRs through bioinformatic analyses of public datasets.

**Methods:** This study retrieved the microarray data sets of GSE106792 and GSE77939 from the Gene Expression Omnibus (GEO) database. After merging two microarray data and adjusting the batch effect, differentially expressed genes (DEGs) were identified. Gene Ontology (GO) resource and Kyoto Encyclopedia of Genes and Genomes (KEGG) resource were conducted to analyze the biological process and functional enrichment. We performed receiver operating characteristic (ROC) curves to filtrate potential diagnostic markers for INRs. Gene Set Enrichment Analysis (GSEA) was conducted to perform the pathway enrichment analysis of individual genes. Single sample GSEA (ssGSEA) was performed to assess scores of immune cells within INRs and IRs. The correlations between the diagnostic markers and differential immune cells were examined by conducting Spearman’s rank correlation analysis. Subsequently, miRNA-mRNA-TF interaction networks in accordance with the potential diagnostic markers were built with Cytoscape. We finally verified the mRNA expression of the diagnostic markers in clinical samples of INRs and IRs by performing RT-qPCR.

**Results:** We identified 52 DEGs in the samples of peripheral blood mononuclear cells (PBMC) between INRs and IRs. A few inflammatory and immune-related pathways, including chronic inflammatory response, T cell receptor signaling pathway, were enriched. FAM120AOS, LTA, FAM179B, JUN, PTMA, and SH3YL1 were considered as potential diagnostic markers. ssGSEA results showed that the IRs had significantly higher enrichment scores of seven immune cells compared with IRs. The miRNA-mRNA-TF network was constructed with 97 miRNAs, 6 diagnostic markers, and 26 TFs, which implied a possible regulatory relationship.

**Conclusion:** The six potential crucial genes, FAM120AOS, LTA, FAM179B, JUN, PTMA, and SH3YL1, may be associated with clinical diagnosis in INRs. Our study provided new insights into diagnostic and therapeutic targets.

## Introduction

Acquired immunodeficiency syndrome (AIDS) refers to a serious chronic infectious disease attributed to the human immunodeficiency virus (HIV). Antiretroviral therapy (ART) has been the most common method for treating AIDS. ART is capable of inhibiting HIV replication, restoring the number of CD4^+^ T cells, and reducing AIDS morbidity and mortality effectively (Saksena et al., 2007; Sabin and Lundgren, 2013).

However, in nearly 15–30% of the infected patients, the number of CD4^+^ T cells has been at a low level for a long time even after HIV is overall suppressed after long-term ART. These patients are termed immunological non-responders (INRs) (Gazzola et al., 2009; Corbeau and Reynes, 2011). Compared with the immunological responders (IRs), the INRs have elevated levels of immune activation, inflammation, and autoantibodies. To be specific, the morbidity and mortality of non-AIDS-related diseases (e.g., cardiovascular disease, non-AIDS-related tumors, and HIV-related neurocognitive disorders) significantly increased (Engsig et al., 2014; Takuva et al., 2014; Pacheco et al., 2015). Therefore, the early diagnosis of INR is imperative for the prevention and treatment of patients’ clinical diseases. At present, the judgment of immune non-response mainly depends on the count of CD4 while the indicator of CD4 count has some defects. If there is no uniform standard for the specific definition of the CD4 count in immunologically non-responsive patients, it is impossible to completely distinguish between INR and IR. Therefore, it is urgent to explore the diagnostic biomarkers of INR, so as to lay a foundation for elucidating the mechanism of immune non-response and clinical diagnosis.

The occurrence of immune non-response in HIV infected persons might be correlated with numerous factors (e.g., age, duration of HIV infection (Baker, et al., 2008a), CD4^+^ T cell counts nadir (Moore and Keruly, 2007), inflammation and adverse reaction to IL-7). As reported in existing studies, INRs achieve high levels of T cell activation, which is defined as the co-expression of CD38 and HLA-DR on CD4^+^ and CD8^+^ T cells and the up-regulated expression of inflammatory markers in plasma (Lederman et al., 2011). However, INRs diagnostic markers have been rarely studied.

Hence, based on the samples of IRs and INRs obtained from the GEO database, several bioinformatics methods are employed to obtain diagnostic markers that can assess immune response and immune non-response. This can be considered an attempt to present novel auxiliary targets for defining immune non-response.

## Materials and Methods

### Data Source and Preprocessing

Raw files of two registered microarray data sets, i.e., GSE106792 and GSE77939, originated from the NCBI GEO database (https://www.ncbi.nlm.nih.gov/geo/). A total of 12 INRs and 12 IRs samples of peripheral blood mononuclear cells (PBMC) were covered in GSE106792 data sets. INR subjects were defined as having CD4^+^ T cell counts below 350 cells/μl, and IRs were defined as possessing CD4^+^ T cell counts above 350/μL after at least 2 years of ART with virologic control. GSE77939 data sets involved 7 INRs and 5 IRs samples of PBMC. IR subjects were defined as undergoing ART for at least one year or more and showing signs of response to the treatment with an increase of over 150 cells/μL in CD4^+^ T cell counts and current CD4 counts above 250 cells/μL. Moreover, INRs included HIV-positive individuals on ART for at least one year or more showing undetectable viral load (<40 copies/mL) and CD4^+^ T cell counts below 250 cells/μL. The combat function in the sva package was adopted to remove the batch effects while normalizing and merging the mentioned two data sets (Leek et al., 2012). Principal component analysis (PCA) was conducted to visualize the spatial distribution of the samples and examine the results of the treatment of the batch effects (Jolliffe and Cadima, 2016).

### Patients and Collection of Clinical Samples

The study subjects comprised two populations, i.e., 20 HIV-1-infected patients on ART with undetectable viremia (HIV-RNA < 50 copies/mL) more than 2 years, consisting of 10 HIV-IRs (with CD4^+^ T cell counts above 350 cells/μL) and 10 HIV-INRs (with CD4^+^ T cell counts below 250 cells/μL) in the sexually transmitted disease (STD) and AIDS Clinic, Beijing Youan Hospital, Capital Medical University. The exclusion criteria included the coinfection with HBV and HCV, pregnancy, as well as moribund status. Specific information on the patients is listed in Supplementary Table S1.

### Identification of Differentially Expressed Genes

The DEGs were determined between INRs and IRs samples with the “limma” R package (Ritchie et al., 2015; Phipson et al., 2016). The |log_2_ fold change (FC)| > 0.5 and adj. *p*-Value < 0.05 were considered the cut-off criteria. The results of DEGs were introduced to the heatmap and the volcano map. The location of DEGs on chromosomes was illustrated with the “OmicCircos” package from R software (Zhu et al., 2020).

### Functional and Pathway Enrichment Analyses of Differentially Expressed Genes

Gene Ontology (GO) enrichment analysis and Kyoto Encyclopedia of Genes and Genomes (KEGG) pathway analysis were conducted with the DEGs by adopting the “clusterProfiler” package (Yu et al., 2012). On the whole, GO enrichment analysis expresses the biological processes (BP), cellular components (CC), and molecular functions (MF) correlated with DEGs. The biological pathways correlated with DEGs were revealed from KEGG pathway analysis. The threshold for enrichment significance was *p*-value < 0.05.

### Screening of the Diagnostic Markers

After the expression data of the DEGs were extracted from the batch-processed data set, the receiver operating characteristic (ROC) curves of the mentioned DEGs were plotted with the pROC in R package Genes. To be specific, the AUC values over 0.9 showed diagnostic significance and could be considered the diagnostic markers (Obuchowski and Bullen, 2018; Cao and López-de-Ullibarri, 2019). The expression of the diagnostic markers in the merged GEO series was drawn into a box plot and the difference was compared by the Wilcoxon test. The *p*-value < 0.05 was defined to be statistically significant.

### Gene Set Enrichment Analysis

To identify the pathways and processes differently activated or suppressed by the diagnostic markers, GSEA was performed for the single diagnostic marker in GSEA software (v3.0). The expression values of the respective diagnostic marker acted as the phenotype files, and the correlation coefficients of the respective diagnostic marker with each gene in the gene sets were ranked. Furthermore, the “h.all.v7.4.symbols.gmt (hallmarks)” and “c7.all.v7.4.symbols.gmt (Immunologic signatures)” from Molecular Signatures Database (MSigDB) (Xiao et al., 2021) were adopted as the reference gene sets. The threshold for enrichment significance was NOM *p*-value < 0.05.

### Evaluation of Immune Cell Infiltration

The enrichment scores of 28 immune cells in the respective sample were determined with single-sample gene set enrichment analysis (ssGSEA) (Bindea et al., 2013). A box plot was generated to visualize the differences in 28 immune cells infiltration between the INRs and the IRs. By adopting ‘Spearman’ correlation analysis, the correlations between the diagnostic markers and the differential immune cells were analyzed. The correlation coefficient |cor| > 0.3 and *p*-value < 0.05 was considered with statistical significance.

### Construction of miRNA-mRNA-TF Regulatory Network

Target miRNAs of the diagnostic markers were estimated according to miRWalk and miRDB databases. The miRNAs identified in both two databases were considered the target miRNAs. The target TFs of the diagnostic markers were predicted by Network Analyst database. Subsequently, the miRNA-mRNA, mRNA-TF, and miRNA-mRNA-TF interaction networks were built with Cytoscape (Li et al., 2020).

### RNA Extraction and Quantitative Real-Time Polymerase Chain Reaction

Total RNA from the 10 INRs and 10 IRs samples was extracted by adopting Nuclezol LS RNA Isolation Reagent following the manufacturer’s instructions (ABP Biosciences Inc.). Subsequently, total RNA was reversely transcribed into cDNA with the SureScript-First-strand-cDNA-synthesis-kit (GeneCopoeia) by complying with the manufacturers’ protocol. qPCR was subsequently performed with the BlazeTaq™ SYBR^®^ Green qPCR Mix 2.0 (GeneCopoeia). The thermocycling conditions below were employed for qPCR, 1 cycle at 95°C for 30 s (initial denaturation), followed by 40 cycles of 10 s at 95°C (denaturation), 20 s at 60°C (annealing), and 30 s at 72°C (extension). Table 1 lists the sequences of the primers. The relative expression level was normalized to the endogenous control GAPDH and then calculated by applying the 2^−ΔΔCq^ method (Livak and Schmittgen, 2001). The student’s t-test was performed to compare the differences between the two groups. The two-tailed *p*-value < 0.05 in the statistical analysis was defined to be statistically significant.

**TABLE 1:** The relationship in the miRNA-mRNA-TF regulatory network.

Genes	Sequence
FAM120AOS	
Forword	5′-TGG​GAA​GGG​GGA​TAG​GGG-3’
Reverse	5′-GTC​AAG​CGG​CAG​GGC​AAC-3’
LTA	
Forword	5‘-CAG​GTG​GTC​TTC​TCT​GGG​AAA-3’
Reverse	5‘-GTG​TGG​GTG​GAT​AGC​TGG​TCT-3’
FAM179B	
Forword	5′-AGG​CCG​TCG​AAG​AAC​TAA​A-3’
Reverse	5′-CTC​CAA​GGC​GAA​TAA​CCA​G-3’
JUN	
Forword	5′-TGC​CTC​CAA​GTG​CCG​AAA-3’
Reverse	5′-GCT​GTG​CCA​CCT​GTT​CCC-3’
PTMA	
Forword	5′-CAC​CAG​CTC​CGA​AAT​CAC-3’
Reverse	5′-TCC​TCC​TCC​TCT​TCC​TCC-3’
SH3YL1	
Forword	5′-GCA​CCA​GTC​CAG​CTG​AAC​TCT-3’
Reverse	5′-TTC​CTT​CCC​ACC​AAT​CAA​AAT-3’
GAPDH	
Forword	5′-CGC​TGA​GTA​CGT​CGT​GGA​GTC-3’
Reverse	5′-GCT​GAT​GAT​CTT​GAG​GCT​GTT​GTC-3’

## Results

### Identification of Differentially Expressed Genes

**FIGURE 1:**
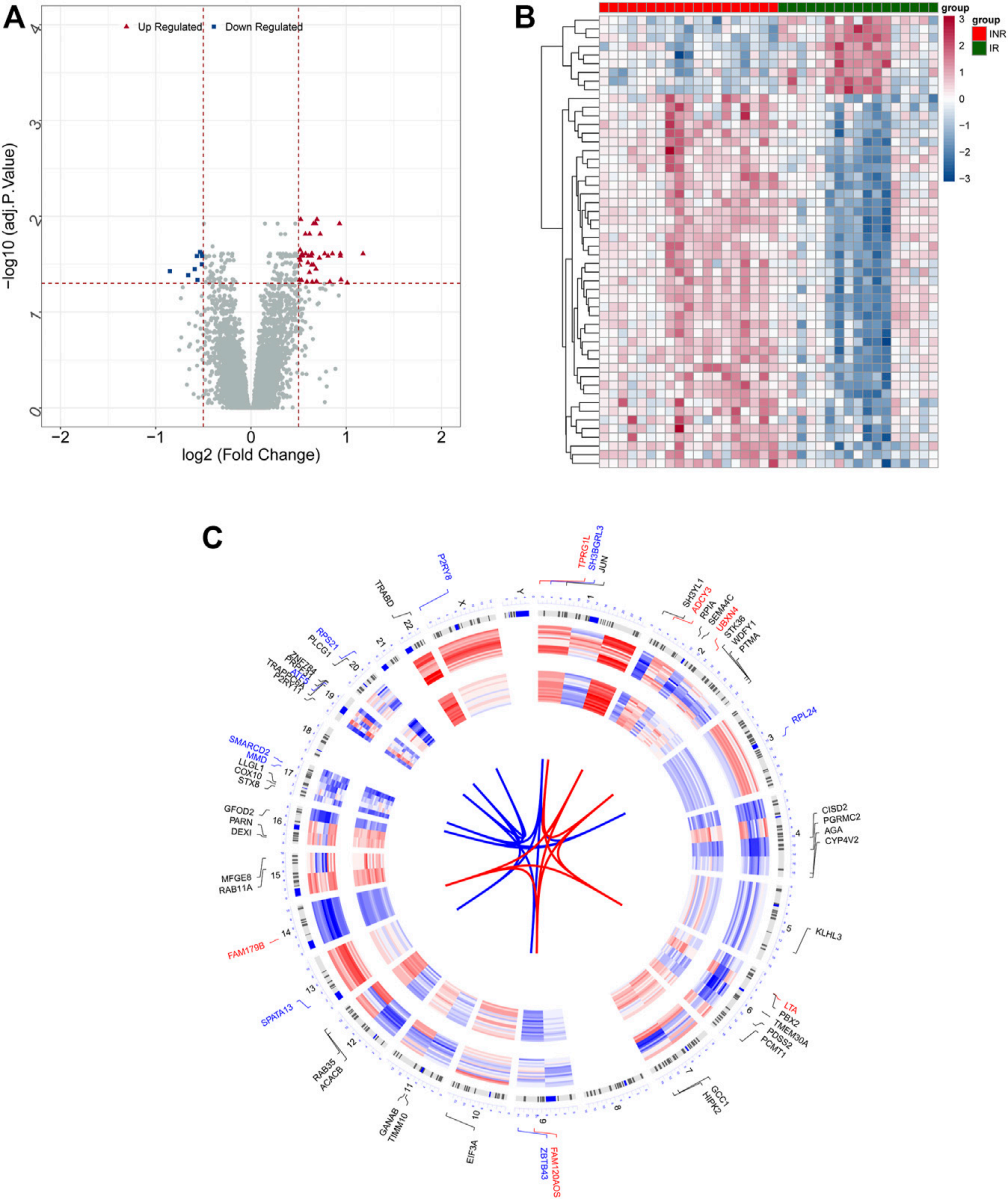
Identification of DEGs between INRs and 17 IRs. **(A,B)** Volcano plot **(A)** and Heatmap **(B)** presented the expression of DEGs. **(C)** The chromosomal locations and expression patterns of DEGs.

Gene expression levels of merged GEO series with batch effects adjusted were standardized, and the results of PCA before and after processing are presented in Supplementary Figures S1A–C. Merged data sets covered 19 INRs and 17 IRs samples. In total 52 DEGs with |log_2_ FC| > 0.5 in the INRs samples compared with the IRs samples were identified, i.e., 43 up-regulated genes and 9 down-regulated genes (Supplementary Table S2). Figures 1A,B presents the heatmap plot and volcano plot of 52 DEGs recruited in subsequent analyses. Moreover, the chromosomal locations and expression patterns of the mentioned DEGs are presented in Figure 1C.

### Functional Enrichment Analyses of Differentially Expressed Genes

**FIGURE 2:**
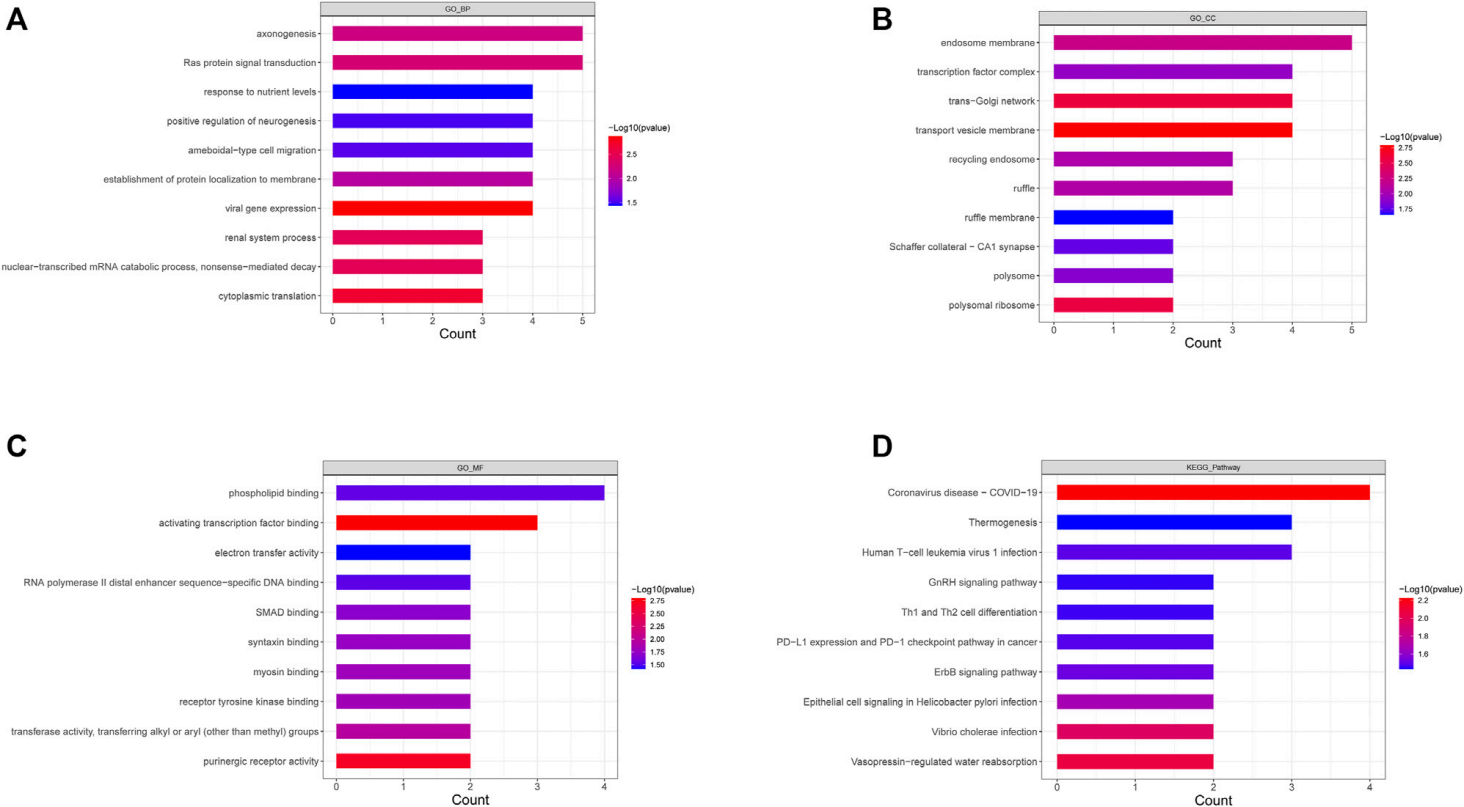
Go annotation an KEGG enrichment analysis of DEGs. **(A–C)** The top 10 terms of BP **(A)**, CC **(B),** and MF **(C)** of GO annotation were enriched by DEGs. **(D)** The top 10 KEGG pathways enriched by DEGs.

To explore the biological functions of the 52 DEGs in-depth, functional enrichment analyses were conducted, and the results are listed in Supplementary Table S3. GO annotation of DEGs consisted of three parts (i.e., BP, CC, and MF), and the top 10 significantly enriched pathways in accordance with the *p*-value of the respective category are illustrated (Figures 2A–C). Inflammatory and immune-related pathways were enriched in the BP ontology (e.g., positive regulation of monocyte, regulation of humoral immune response mediated by circulating immunoglobulin, positive regulation of inflammatory response to an antigenic stimulus, chronic inflammatory response, positive regulation of humoral immune response, toll-like receptor three signaling pathways). In the CC ontology, the DEGs were significantly correlated with the endosome membrane, transcription factor complex, trans-Golgi network, transport vesicle membrane, etc. For MF, the DEGs were significantly correlated with phospholipid binding, activating transcription factor binding, electron transfer activity, etc. Next, KEGG analysis was conducted to investigate the vital pathways involved, and the results are displayed in Supplementary Table S4. Figure 2D lists the top 10 enriched terms based on *p*-values. It was reported that inflammatory and immune-related pathways were enriched (e.g., Th1 and Th2 cell differentiation, inflammatory mediator regulation of TRP channels, NF-kappaB signaling pathway, T cell receptor signaling pathway, Th17 cell differentiation, and Th17 cell differentiation).

### Identification of the Diagnostic Markers and Gene Set Enrichment Analysis

**FIGURE 3:**
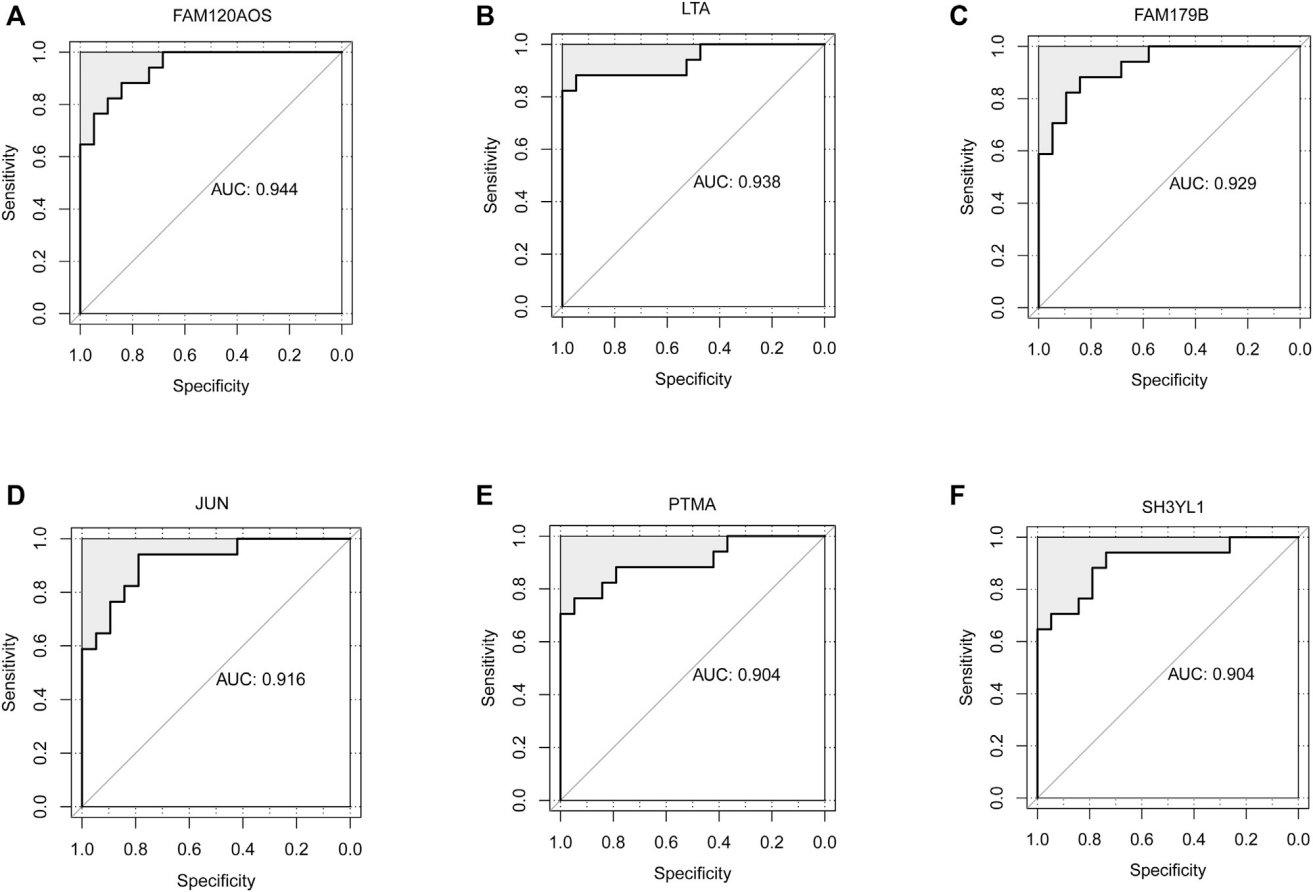
Identification of diagnostic markers by ROC analysis. **(A–F)** ROC curves showed the AUC values of FAM120AOS **(A)**, LTA **(B)**, FAM179B **(C)**, JUN **(D)**, PTMA **(E)**, SH3YL1 **(F)**.

**FIGURE 4:**
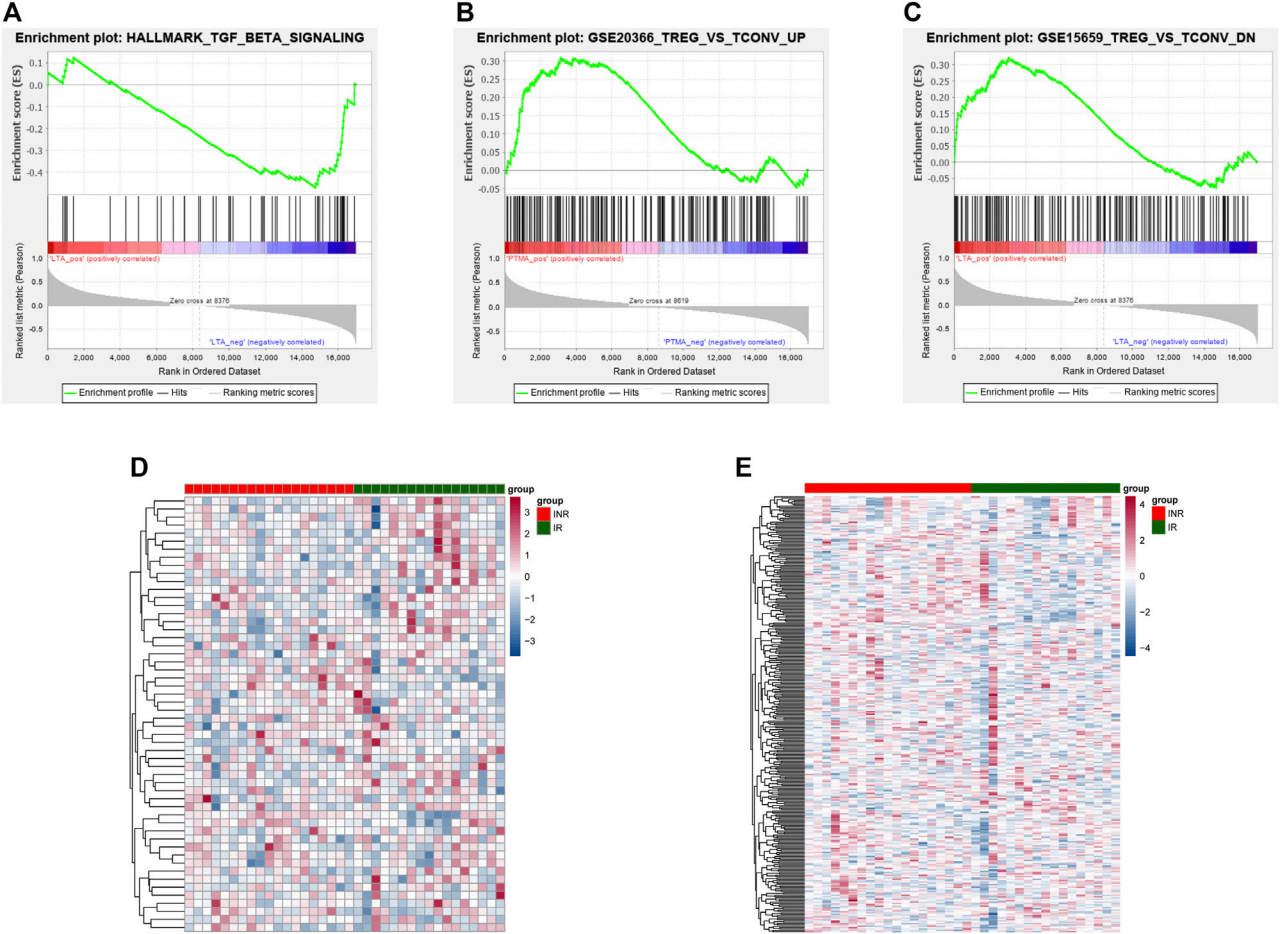
The results of enrichment analyses of diagnostic markers. **(A)** HALLMARK_TGF_BETA_SIGNALING pathway enriched by LTA. **(B–C)** TREG_VS_TCONV_UP and TREG_VS_TCONV_DN pathway enriched by PTMA **(B)** and LTA **(C)**. **(D)** The expression of genes in the HALLMARK_TGF_BETA_SIGNALING pathway between INRs and IRs. **(E)** The expression of genes in TREG_VS_TCONV_UP and TREG_VS_TCONV_DN pathway between INRs and IRs.

ROC analysis was conducted on the 52 DEGs and excavated six genes (*FAM120AOS, LTA, FAM179B, JUN, PTMA, SH3YL1*) with AUC values over 0.9 (Figures 3A–F), thereby illustrating that the mentioned genes exhibited a powerful discrimination ability to discriminate INRs samples from the IRs samples. GSEA analysis is applicable to the pathway enrichment analysis of individual genes. Subsequently, GSEA was conducted for a single diagnostic marker in the expression data of merged data set in accordance with hallmark gene sets and immunologic signature gene sets (Xiao, et al., 2021). Supplementary Table S5 presents the enrichment results of hallmark and immunologic signature function terms for the respective diagnostic marker. Given NOM *p*-value, inflammatory signaling pathway, HALLMARK_TGF_BETA_SIGNALING was significantly correlated with LTA expression (Figure 4A). Treg cell-related terms: TREG_VS_TCONV_UP and TREG_VS_TCONV_DN were significantly correlated with PTMA and LTA expressions, respectively (Figures 4B,C). Thus, the genes involved in the mentioned three gene sets(HALLMARK_TGF_BETA_SIGNALING, TREG_VS_TCONV_UP, TREG_VS_TCONV_DN) were obtained, and their expression patterns in the INRs and IRs groups were demonstrated with heatmaps (Younes et al., 2018) (Figures 4D,E).

### Comparison of the Immune Microenvironment in INRs and IRs Samples

**FIGURE 5:**
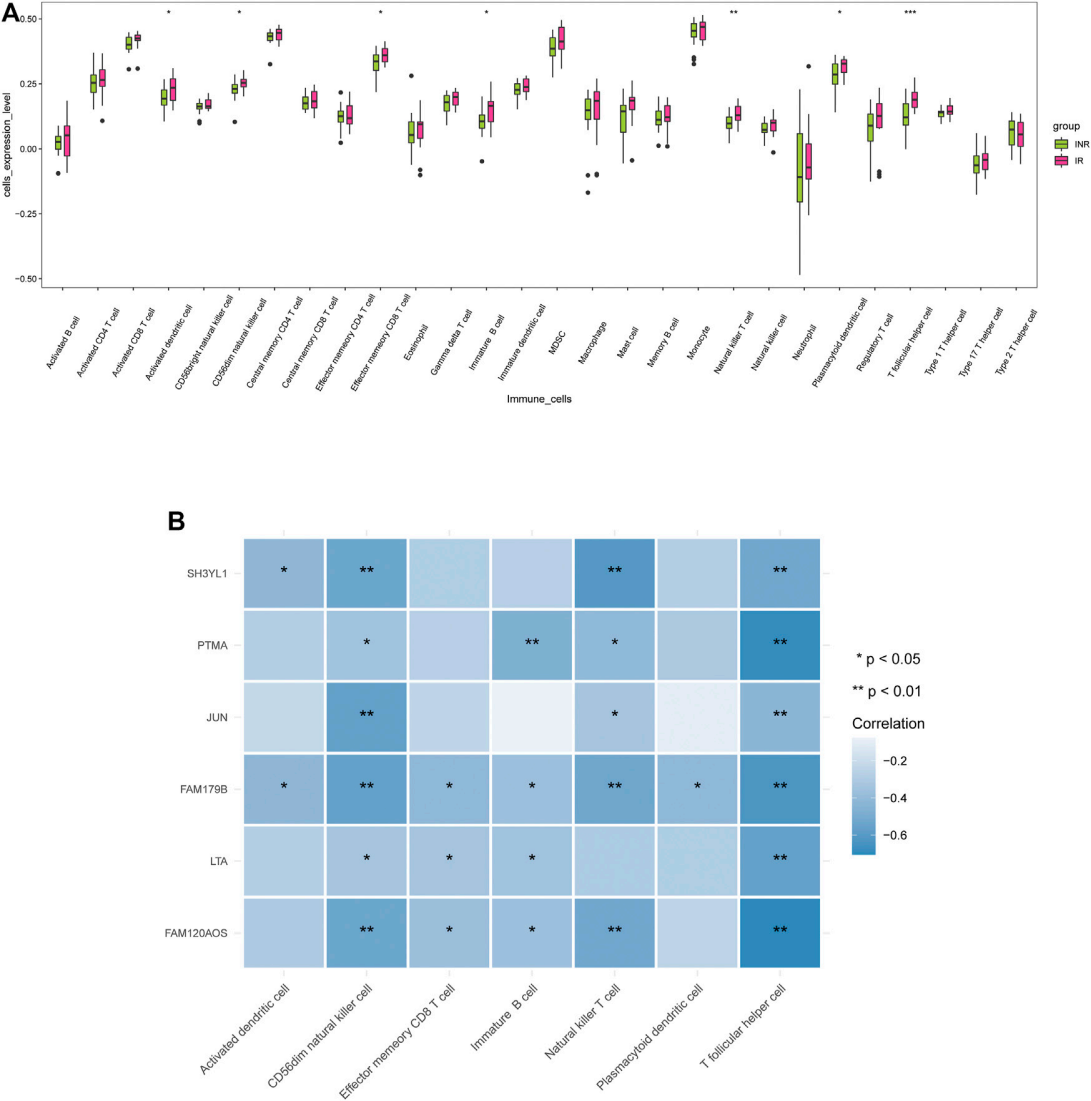
Correlations between diagnostic markers and immune cells. **(A)** The discrepancy of enrichment scores for 28 immune cells between the INRs and IRs samples. **(B)** The correlation heatmap of 6 diagnostic markers and 7 differential immune cells.

To assess the discrepancy of immune microenvironment between the INRs and IRs samples, the enrichment scores of 28 immune cells were estimated by employing the ssGSEA algorithm. As indicated from the results, the IRs achieved significantly higher enrich scores of activated dendritic cell, CD56^dim^ natural killer (NK) cell, effector memory CD8 T cell, immature B cell, natural killer T cell, plasmacytoid dendritic cell, as well as T follicular helper cell (Figure 5A). As revealed from the mentioned findings, the difference of immune cells can be inferred that there are significant differences in immune microenvironment between INRs and IRs.

The spearman correlation between six diagnostic markers and differential immune cells between INRs and IRs was further analyzed (Supplementary Table S6). According to Figure 5B, all six diagnostic markers were negatively correlated with T follicular helper cell and CD56^dim^ NK cell. SH3YL1 and FAM179B were negatively correlated with activated dendritic cell; FAM179B, LTA, and FAM120AOS were negatively correlated with effector memory CD8 T cell; PTMA, FAM179B, LTA, and FAM120AOS were negatively correlated with immature B cell; SH3YL1, PTMA, JUN, FAM179B and FAM120AOS were negatively correlated with natural killer T cell; FAM179B was negatively correlated with plasmacytoid dendritic cell.

### miRNA-mRNA-TF Regulatory Network Analysis

**FIGURE 6:**
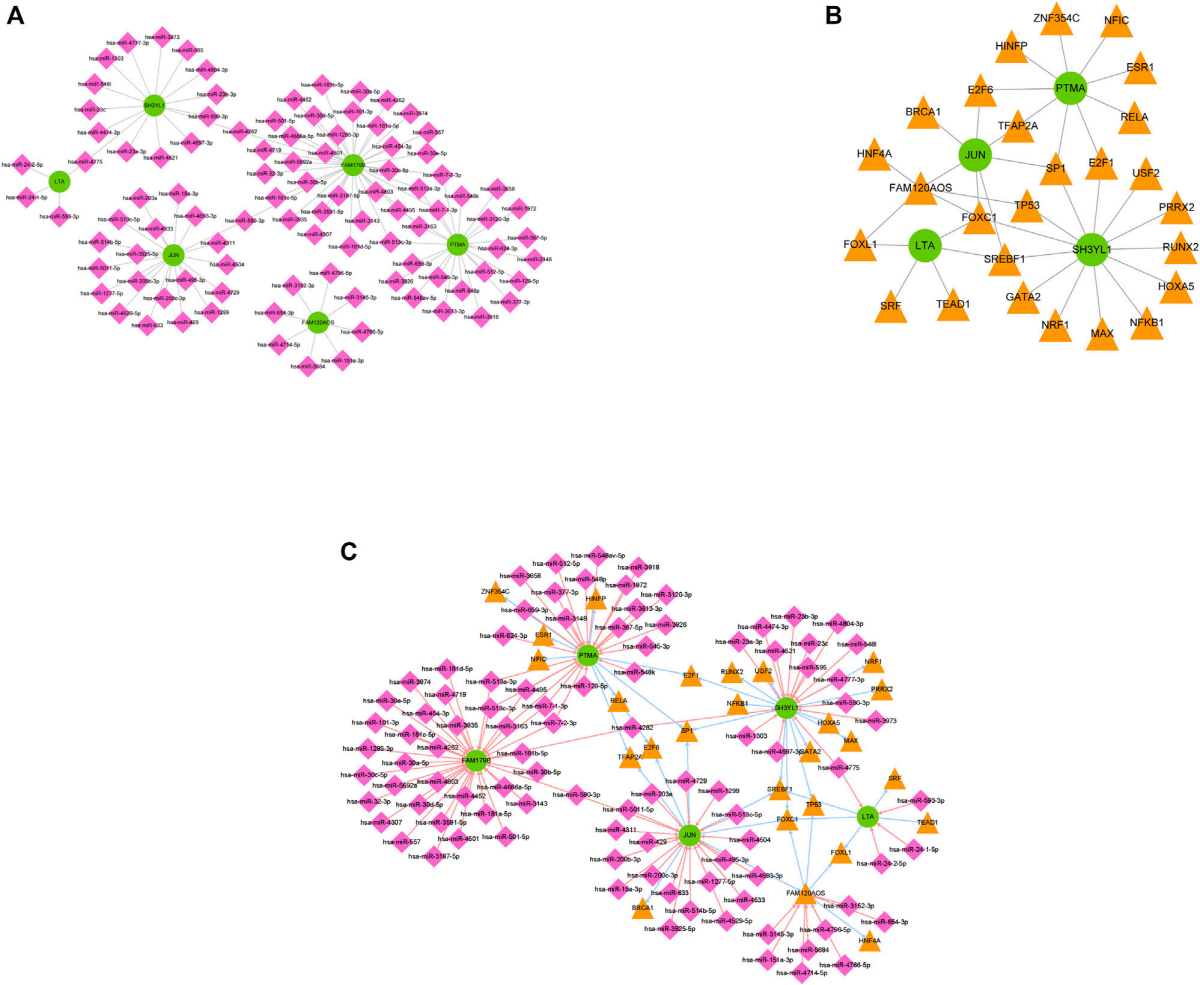
The regulating mechanisms of diagnostic markers. **(A)** The miRNA mRNA regulatory network comprising 97 miRNAs, 6 diagnostic markers, and 106 edges. **(B)** The mRNA-TF regulatory network comprising 38 mRNA TF pairs. **(C)** The miRNA-mRNA-TF network of 6 diagnostic markers.

The regulated networks have been recognized to critically help understand the mechanisms of disease. To explore the regulatory mechanisms involved in INRs in-depth, miRNAs targeting the diagnostic markers were estimated by the miRDB and miRWalk databases. On the whole, 97 putative miRNAs and 106 miRNA-mRNA pairs were identified (Supplementary Table S7). A miRNA-mRNA regulatory network was built with 97 miRNAs, six diagnostic markers, and 106 edges (Figure 6A). Likewise, mRNA and TF-mediated regulatory networks were built as well. In general, 26 TFs were predicted by using the Network Analyst database, and the mRNA-TF regulatory network consisted of 38 mRNA-TF pairs (Figure 6B, Supplementary Table S8). Given the predicted miRNA-mRNA and mRNA-TF networks above, the miRNA-mRNA-TF network was built by applying Cytoscape software and displayed in Figure 6C. The detailed information of Figure 6C was listed in Table 2. In such a regulatory network, PTMA, SH3YL1, and JUN were targeted by Sp1 transcription factor (SP1), SH3YL1 was targeted by nuclear factor kappa B subunit 1 (NFKB1), and PTMA and JUN were targeted by transcription factor AP-2 alpha (TFAP2A). Furthermore, FAM179B was regulated by hsa-miR-101-3p, and FAM179B was regulated by hsa-miR-24-1-5p and hsa-miR-24-2-5p, probably correlated with HIV progression.

**TABLE 2:** The relationship in the miRNA-mRNA-TF regulatory network.

mRNA	TF	miRNA
FAM120AOS	HNF4A	hsa-miR-3152-3p
	FOXL1	hsa-miR-654-3p
	TP53	hsa-miR-3145-3p
	FOXC1	hsa-miR-5684
		hsa-miR-4714-5p
		hsa-miR-151a-3p
		hsa-miR-4796-5p
		hsa-miR-4766-5p
JUN	E2F6	hsa-miR-513c-5p
	TFAP2A	hsa-miR-3925-5p
	SP1	hsa-miR-429
	SREBF1	hsa-miR-580-3p
	FOXC1	hsa-miR-200b-3p
	FAM120AOS	hsa-miR-200c-3p
	BRCA1	hsa-miR-203a
		hsa-miR-4311
		hsa-miR-4729
		hsa-miR-633
		hsa-miR-4504
		hsa-miR-514b-5p
		hsa-miR-5011-5p
		hsa-miR-15a-3p
		hsa-miR-1299
		hsa-miR-4529-5p
		hsa-miR-4533
		hsa-miR-495-3p
		hsa-miR-1277-5p
		hsa-miR-4693-3p
LTA	FOXL1	hsa-miR-593-3p
	SRF	hsa-miR-4775
	TEAD1	hsa-miR-24-1-5p
	SREBF1	hsa-miR-24-2-5p
	FOXC1	
PTMA	NFIC	hsa-miR-3120-3p
	ESR1	hsa-miR-548p
	RELA	hsa-miR-3658
	E2F1	hsa-miR-548av-5p
	SP1	hsa-miR-7-2-3p
	TFAP2A	hsa-miR-1972
	E2F6	hsa-miR-3613-3p
	HINFP	hsa-miR-624-3p
	ZNF354C	hsa-miR-3163
		hsa-miR-377-3p
		hsa-miR-512-5p
		hsa-miR-3926
		hsa-miR-4495
		hsa-miR-513a-3p
		hsa-miR-548k
		hsa-miR-513c-3p
		hsa-miR-7-1-3p
		hsa-miR-129-5p
		hsa-miR-545-3p
		hsa-miR-3918
		hsa-miR-367-5p
		hsa-miR-659-3p
		hsa-miR-3148
SH3YL1	TP53	hsa-miR-23a-3p
	SP1	hsa-miR-23b-3p
	E2F1	hsa-miR-1303
	USF2	hsa-miR-23c
	PRRX2	hsa-miR-595
	RUNX2	hsa-miR-548l
	HOXA5	hsa-miR-4282
	NFKB1	hsa-miR-590-3p
	MAX	hsa-miR-4775
	NRF1	hsa-miR-4804-3p
	GATA2	hsa-miR-4777-3p
	SREBF1	hsa-miR-3973
	FOXC1	hsa-miR-4521
		hsa-miR-4474-3p
		hsa-miR-4697-3p
FAM179B		hsa-miR-4666a-5p
		hsa-miR-3187-5p
		hsa-miR-4282
		hsa-miR-501-5p
		hsa-miR-5692a
		hsa-miR-30a-5p
		hsa-miR-4719
		hsa-miR-7-1-3p
		hsa-miR-30b-5p
		hsa-miR-513c-3p
		hsa-miR-3143
		hsa-miR-557
		hsa-miR-30c-5p
		hsa-miR-3935
		hsa-miR-101-3p
		hsa-miR-1285-3p
		hsa-miR-30d-5p
		hsa-miR-32-3p
		hsa-miR-4307
		hsa-miR-454-3p
		hsa-miR-4262
		hsa-miR-30e-5p
		hsa-miR-4495
		hsa-miR-3974
		hsa-miR-3163
		hsa-miR-7-2-3p
		hsa-miR-181a-5p
		hsa-miR-4501
		hsa-miR-181b-5p
		hsa-miR-4452
		hsa-miR-4803
		hsa-miR-580-3p
		hsa-miR-181c-5p
		hsa-miR-3591-5p
		hsa-miR-513a-3p
		hsa-miR-181d-5p

### Validation of the Expression of the Diagnostic Markers Through RT-qPCR

**FIGURE 7:**
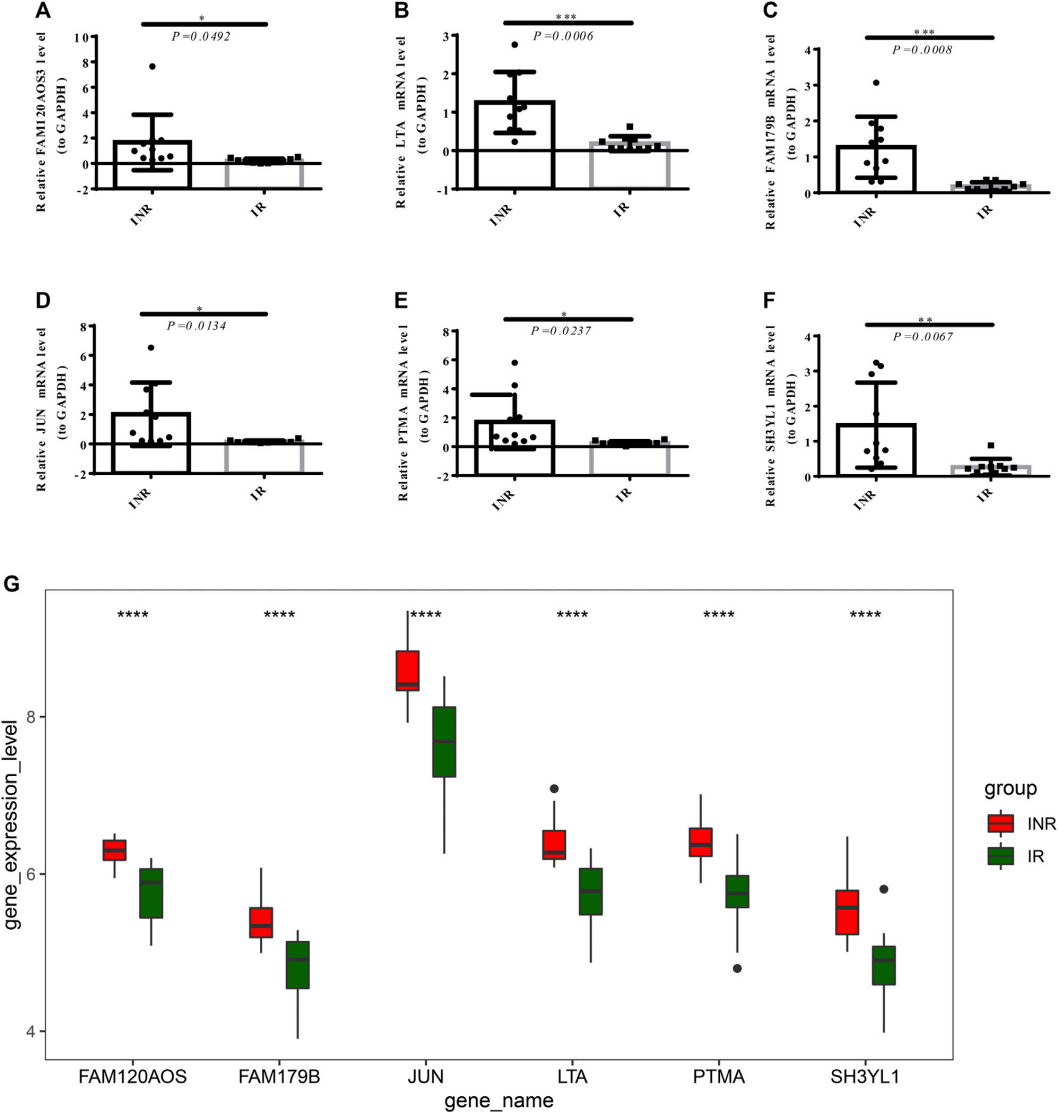
The mRNA expression of diagnostic markers in clinical 10 INRs and 10 IRs samples were detected by RT-PCR. The expression of FAM120AOS, LTA, FAM179B, JUN, PTMA, and SH3YL1 **(A–F)** between the INRs and IRs samples (*p < 0.05, **p < 0.01, ***p < 0.001). **(G)** The expression of the diagnostic markers in merged GEO series (****p < 0.0001).

The mRNA expression of the diagnostic markers in clinical 10 INRs and 10 IRs samples were detected by performing RT-qPCR. As revealed in Figures 7A–F, the expressions of FAM120AOS, LTA, FAM179B, JUN, PTMA, and SH3YL1 were significantly up-regulated in INRs compared with IRs, thereby further verifying the expression of the diagnostic markers in the merged data sets from the public database (Figure 7G).

## Discussion

Although ART can effectively inhibit HIV replication (Bai et al., 2020) and promote the recovery of immune function of infected persons, there are still 20–30% of HIV-infected persons (Li et al., 2011). Moreover, the number of CD4^+^ T cells remains at a low level (<350 cells/μL) for a long time even after the virus is completely suppressed (Yang et al., 2020). This phenomenon is called poor immune reconstitution. Such patients are called INRs. Compared with IRs, the incidence and mortality of INRs non-AIDS-related diseases (such as cardiovascular diseases, tumors, and neurocognitive disorders) and AIDS-related diseases (such as infections and tumors) have increased significantly, resulting in an increase in the medical and economic burden on the family and society (Baker, et al., 2008b; Liu et al., 2021; Pacheco, et al., 2015; Young et al., 2012). However, there is still a lack of biomarkers for early prediction or diagnosis of immune non-response. Thus, early prediction or diagnostic biomarkers of INR should be explored urgently to provide a foundation for clinical diagnosis of INR, so as to improve the immune reconstitution status of HIV-infected patients.

In the present study, we firstly identified 52 differentially expressed genes in the samples of immune non-responders and immune responders of HIV-infected individuals. As revealed from the subsequent functional enrichment analysis, the mentioned genes were largely involved in inflammatory and immune response-related pathways. (Figures 2A,D). To be specific, the higher level of inflammatory response and immune activation of INRs primarily accounts for why they are more likely to develop AIDS than IRs after ART treatment (Lu et al., 2018). For instance, sCD14, a marker of acute phase response, monocyte and macrophage activation, was reported to be significantly elevated in INRs (Ruiz-Briseño et al., 2020). In addition, sCD163 levels, another marker of monocyte activation and inflammatory response, were noticeably regulated after the treatment with ART (McKibben et al., 2015). More importantly, higher immune activation and systemic inflammatory biomarkers (sST2 and hsCRP) levels were found to be directly correlated with the presence of arterial hypertension and diastolic dysfunction in INRs (Scherzer et al., 2018). Besides, Hunt et al. reported that for every 5% increase in the percentage of activated CD4^+^ T cells, the number of CD4^+^ T cells decreased by 45 cells/µl in the first 3 months of antiretroviral therapy. Likewise, for each 5% increase in the percentage of activated CD8^+^ T cells, CD4^+^ T cells decreased by 35 cells/µl (Hunt et al., 2003). Furthermore, plasma levels of sCD14 and sCD163 and activated CD4^+^ and CD8^+^ T ratio was significantly higher in HIV-infected individuals on ART compared with the healthy controls (Cobos Jiménez et al., 2016). Accordingly, the aforementioned differentially expressed 52 genes might be vital molecules in the regulation of immune activation and inflammatory response, probably acting as markers of immune non-response after ART treatment.

Moreover, as demonstrated by ROC analysis, *FAM120AOS*, *LTA*, *FAM179B*, *JUN*, *PTMA,* and *SH3YL1* could act as the markers for the diagnosis of INRs (Figures 3A–F). It is gratifying that LTA in the mentioned six markers were significantly enriched in the TGF-Beta signaling pathway and immune signaling pathway (TREGVSTCONVUP, TREGVSTCONVDN), and PTMA was involved in the mentioned two immune-related signaling pathways as well (Figures 4A–C). Interestingly, FAM120AOS was suggested to be able to regulate the expression of ITGB1 (CD29) (Tao et al., 2006), whereas ITGB1 has already been reported to be correlated with HIV-1 infection (Diagbouga et al., 2001; Martin-Jaular et al., 2021). In addition, ITGB1 has been found to be involved in T cell apoptosis (Fukumori et al., 2003). LTA is capable of exhibiting identical inflammatory properties to lipopolysaccharide by interacting with toll-like receptors (TLRs) (Kengatharan et al., 1998; Chang et al., 2010), i.e., eliciting different inflammatory responses in resident cells via different signaling cascades (Akira et al., 2006). More importantly, LTA was indicated to be able to facilitate HIV infection of primary oral cells (Dai et al., 2014). JUN could participate in the process of differentiation of naïve T cells into Th1 and Th2, thereby bringing critical to T cell-mediated diseases (Rincón et al., 1997; Zenz et al., 2008). Next, JUN can stimulate HIV-1 transcription in precursor cells of monocytes and primary macrophages (Varin et al., 2005). Besides, JUN can regulate the inflammatory response by activating antioxidant response elements (Jackson et al., 2011). According to existing studies, exogenous PTMA is capable of effectively inhibiting HIV-1 replication in primary macrophages by regulating some genes that inhibit HIV-1 replication when applied to growth mediators of primary macrophages (Mosoian et al., 2006; Mosoian et al., 2007). As proven by recent studies, SH3YL1 expression can affect T cell activation in multiple sclerosis patients (Fernandes et al., 2019). However, to the extent of our knowledge, there are no studies on FAM179B in relation to immunization or HIV. Thus, this study indicated initially that FAM179B might influence ART treatment in HIV-infected individuals. In brief, this study hypothesized that FAM120AOS, LTA, FAM179B, JUN, PTMA, and SH3YL1 may affect ART treatment by regulating immune or inflammatory responses.

Further, it was found that the mentioned diagnostic genes were correlated with differential immune cells in ART-responsive and non-responsive patients. Particularly, it was demonstrated that CD56^dim^CD16^+^ NK cell subsets are the main subpopulation of peripheral blood NK cells. They express FcγR IIIa (CD16), which makes NK cells cytotoxic (Caligiuri, 2008). Notably, CD56^dim^CD16^+^ subsets have been verified to play a role in HIV pathogenesis. A study revealed that compared to IRs, INRs exhibited more CD56^dim^CD16^dim/−^ NK cells and higher activity levels after ART treatment, suggesting that the increase in CD56^dim^CD16^dim/−^ NK cell subsets might be a negative factor in immune reconstitution (Zhang et al., 2020). Besides, Giuliani et al. discovered that the changes of NK cells in INRs may involve disease progression and impaired CD4^+^ T cell recovery. It indicated an increase in the proportion of regulatory CD56^bright^ NK cell subsets in INRs and a negative correlation with CD4^+^ T cell counts (Giuliani et al., 2017). Furthermore, the activity and functional markers of CD56^dim^CD16^+^ NK cells with immune unresponsiveness increased compared to the healthy control group. In the antiviral treatment of HIV, the activation level of CD56^dim^CD16^+^ NK cells and the increase of functional markers were negatively associated with the CD4^+^ T cell counts (Luo et al., 2017). Therefore, FAM120AOS, LTA, FAM179B, JUN, PTMA, and SH3YL1 may significantly affect ART therapy outcomes by regulating activated dendritic cells, CD56^dim^ NK cells, effector memory CD8 T cells, immature B cells, natural killer T cells, plasmacytoid dendritic cells, and T follicular helper cells. However, in-depth studies should be conducted to determine the mechanism of action of the mentioned genes.

Notably, in the miRNA-mRNA-TF networks, the transcription factor SP1 could regulate the expressions of PTMA, SH3YL1, and JUN. The transcription factor NFKB1 could regulate the expression of SH3YL1, and TFAP2A could regulate the expressions of PTMA and JUN. Consistent with the results here, it was shown that SP1 could enhance JUN expression (Yi et al., 2020). Since other regulatory mechanisms have not been reported, in-depth experiments should be conducted to verify the regulatory mechanisms of the mentioned genes.

Taken together, we have identified six genes associated with immunological non-response in HIV-infected individuals. Single or combination of these genes might be used as diagnostic markers for INRs. Thus, our findings provide some clues for exploring the mechanisms of incomplete immune reconstitution in HIV-infected individuals and may help to guide ART treatment in HIV-infected individuals. However, the expression of these six genes should be verified in larger samples. In addition, to further elucidate the regulatory mechanisms of the mentioned genes, considerable experimental studies should be conducted.

## Data Availability

The original contributions presented in the study are included in the article/Supplementary Material, further inquiries can be directed to the corresponding author.

## Supplementary Material

Supplementary Figure 1 The batch effect correction of the GSE106792 and GSE77939 datasets. **(A)** The distribution of samples before correlation in the GSE106792 and GSE77939 datasets. **(B)** The distribution of samples after correlation in the GSE106792 and GSE77939 datasets. **(C)** The distribution of INRs and IRs after correlation.
