# Supplementary figures and images for: Identification of Diagnostic Markers Correlated With HIV+ Immune Non-response Based on Bioinformatics Analysis
Source: Front Mol Biosci. 2021 Dec 22;8:809085. doi: 10.3389/fmolb.2021.809085 (PMC8727996; doi:10.3389/fmolb.2021.809085)

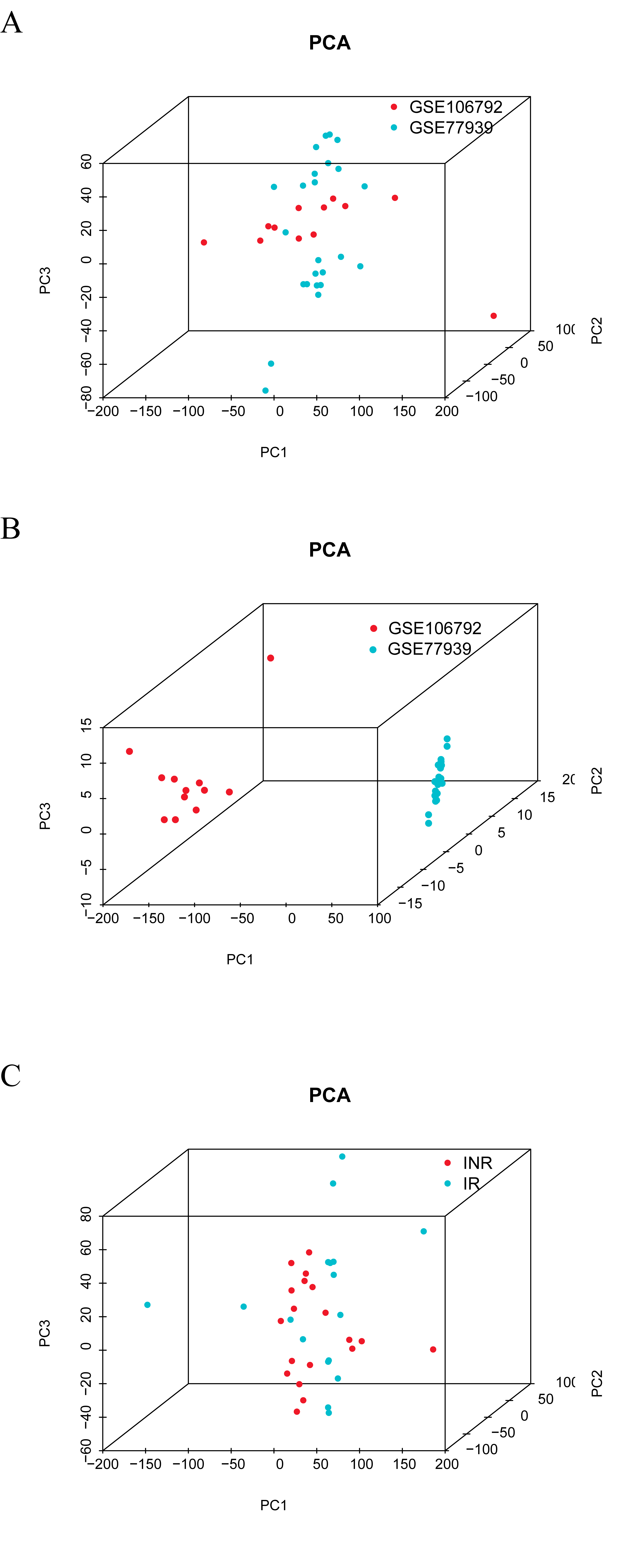

Supplement: Supplementary file 1 [file Image1.tif]
