# Supplementary material for: Identification of Diagnostic Markers Correlated With HIV+ Immune Non-response Based on Bioinformatics Analysis
Source: Front Mol Biosci. 2021 Dec 22;8:809085. doi: 10.3389/fmolb.2021.809085 (PMC8727996; doi:10.3389/fmolb.2021.809085)
Supplement: Supplementary file 2 [file DataSheet1.DOCX]

Supplementary Data

# Supplementary Tables

Supplementary Table 1. Characteristics of HIV-1-infected patients enrolled in the study.

| Patient no. | Gender | Age (y) | Possible transmission route | CD4^+^T  cells/µL | CD8^+^T  cells/µL | CD4/CD8  ratio |
| --- | --- | --- | --- | --- | --- | --- |
| CD4 > 350 (n = 10) | | | | | | |
| P1 | M | 29 | MSM | 655 | 1278 | 0.51 |
| P2 | M | 38 | MSM | 617 | 993 | 0.62 |
| P3 | M | 31 | MSM | 576 | 582 | 0.99 |
| P4 | M | 27 | MSM | 526 | 953 | 0.55 |
| P5 | M | 35 | MSM | 514 | 871 | 0.59 |
| P6 | M | 40 | MSM | 476 | 827 | 0.58 |
| P7 | M | 32 | MSM | 465 | 764 | 0.61 |
| P8 | M | 31 | MSM | 452 | 582 | 0.78 |
| P9 | M | 27 | MSM | 419 | 827 | 0.51 |
| P10 | M | 33 | MSM | 413 | 764 | 0.54 |
| CD4＜250 (n=10) | | | | | | |
| P11 | M | 25 | MSM | 220 | 396 | 0.56 |
| P12 | M | 41 | MSM | 214 | 504 | 0.42 |
| P13 | M | 34 | MSM | 207 | 455 | 0.45 |
| P14 | M | 28 | MSM | 187 | 395 | 0.47 |
| P15 | M | 33 | MSM | 170 | 553 | 0.31 |
| P16 | M | 28 | MSM | 163 | 564 | 0.29 |
| P17 | M | 36 | MSM | 145 | 392 | 0.37 |
| P18 | M | 32 | MSM | 139 | 538 | 0.26 |
| P19 | M | 38 | MSM | 121 | 836 | 0.14 |
| P20 | M | 29 | MSM | 117 | 513 | 0.23 |
| Abbreviations:P,patient; M, male; MSM, men who have sex with men. | | | | | | |

Supplementary Table 2. Identification of 52 DEGs with |log2 FC| > 0.5 in INRs samples compared with IRs samples.

|  | logFC | AveExpr | t | P.Value | adj.P.Val | B |
| --- | --- | --- | --- | --- | --- | --- |
| PBX2 | 1.176251659 | 6.585286404 | 4.654890949 | 4.07E-05 | 0.024641026 | 2.098001317 |
| HIPK2 | 1.010192297 | 6.46333375 | 3.812483762 | 0.000503688 | 0.049712855 | -0.1464754 |
| MFGE8 | 0.94463478 | 6.534191375 | 3.885215846 | 0.000407644 | 0.045963832 | 0.041573958 |
| RAB35 | 0.938810359 | 7.093876035 | 4.467485532 | 7.19E-05 | 0.026058233 | 1.588253345 |
| TMEM30A | 0.938351116 | 5.52594477 | 4.677090837 | 3.80E-05 | 0.024641026 | 2.1586681 |
| JUN | 0.929273425 | 8.112830829 | 5.323876066 | 5.15E-06 | 0.011922097 | 3.943371206 |
| STK36 | 0.854590425 | 6.406712483 | 4.564713258 | 5.35E-05 | 0.024641026 | 1.852157358 |
| PGRMC2 | 0.827893888 | 6.6813675 | 3.843041669 | 0.000460923 | 0.048437788 | -0.067632659 |
| GANAB | 0.808834574 | 6.553311599 | 4.395868279 | 8.94E-05 | 0.026058233 | 1.394717678 |
| LLGL1 | 0.773638182 | 5.871959529 | 4.649085656 | 4.14E-05 | 0.024641026 | 2.082145994 |
| PARN | 0.726560506 | 6.940792398 | 5.012913729 | 1.35E-05 | 0.015354903 | 3.082034742 |
| GCC1 | 0.718135482 | 6.684506281 | 4.305433905 | 0.000117332 | 0.027285348 | 1.151472152 |
| LTA | 0.692670351 | 6.079529075 | 5.773514882 | 1.27E-06 | 0.010760231 | 5.191840095 |
| SEMA4C | 0.691033473 | 5.590393223 | 3.846139942 | 0.000456789 | 0.048437788 | -0.059625291 |
| EIF3A | 0.684642133 | 6.618821559 | 3.832387269 | 0.000475417 | 0.048437788 | -0.095149693 |
| ATF5 | 0.682391085 | 6.08717813 | 4.095177207 | 0.000219798 | 0.035536144 | 0.59153738 |
| SH3YL1 | 0.678979157 | 5.204862936 | 5.22417078 | 7.02E-06 | 0.011922097 | 3.666786485 |
| PTMA | 0.673003942 | 6.092052045 | 5.441943882 | 3.57E-06 | 0.011922097 | 4.271172714 |
| FAM179B | 0.657033588 | 5.118306849 | 5.373649276 | 4.41E-06 | 0.011922097 | 4.081533703 |
| UBXN4 | 0.65631966 | 6.276838378 | 4.165591822 | 0.000178295 | 0.032293618 | 0.778118764 |
| TRAPPC6A | 0.642571902 | 7.194520522 | 3.8283621 | 0.000481008 | 0.04852069 | -0.105537828 |
| KLHL3 | 0.640795035 | 6.066645353 | 4.602838583 | 4.77E-05 | 0.024641026 | 1.955976049 |
| TRABD | 0.638055971 | 7.893967452 | 4.164590445 | 0.000178828 | 0.032293618 | 0.775458432 |
| ADCY3 | 0.636809373 | 7.061810983 | 4.619565107 | 4.53E-05 | 0.024641026 | 2.001579843 |
| ACACB | 0.632854159 | 5.502010916 | 4.378673898 | 9.41E-05 | 0.026058233 | 1.348367966 |
| GFOD2 | 0.615394317 | 5.16804862 | 5.087796132 | 1.07E-05 | 0.015354903 | 3.289057011 |
| WDFY1 | 0.61381825 | 6.331774222 | 4.035694964 | 0.000262091 | 0.038689224 | 0.434725152 |
| RPIA | 0.602078454 | 6.896411775 | 4.501828228 | 6.48E-05 | 0.026058233 | 1.681323001 |
| TPRG1L | 0.597768235 | 7.301601244 | 4.219429904 | 0.000151832 | 0.030618077 | 0.921432202 |
| DEXI | 0.587324162 | 6.89299442 | 3.836005196 | 0.000470447 | 0.048437788 | -0.085808983 |
| AGA | 0.572573189 | 5.241993559 | 4.59142712 | 4.94E-05 | 0.024641026 | 1.924882786 |
| CYP4V2 | 0.571565775 | 5.903130525 | 5.039946925 | 1.24E-05 | 0.015354903 | 3.156734836 |
| TIMM10 | 0.54394268 | 5.910017134 | 4.51373559 | 6.25E-05 | 0.025887933 | 1.713630348 |
| RAB11A | 0.537032159 | 7.272102791 | 4.548074462 | 5.63E-05 | 0.024641026 | 1.806905717 |
| PLCG1 | 0.530918437 | 6.530166485 | 4.354227536 | 0.00010131 | 0.026058233 | 1.282549966 |
| PCMT1 | 0.530386283 | 6.745798128 | 3.868534225 | 0.000427962 | 0.04665698 | -0.001675388 |
| MMD | 0.523967678 | 5.433904486 | 4.377681433 | 9.44E-05 | 0.026058233 | 1.34569406 |
| FAM120AOS | 0.521046427 | 6.045267999 | 5.843176853 | 1.02E-06 | 0.010760231 | 5.385037548 |
| COX10 | 0.517463335 | 6.23350974 | 4.265427337 | 0.000132301 | 0.028794161 | 1.044300931 |
| P2RY11 | 0.516412654 | 5.001009102 | 4.847047659 | 2.26E-05 | 0.022521659 | 2.624783127 |
| PDSS2 | 0.510658983 | 5.701487452 | 4.16448236 | 0.000178885 | 0.032293618 | 0.775171297 |
| ZNF784 | 0.508797899 | 5.171901124 | 4.312208968 | 0.000114967 | 0.027106709 | 1.169648733 |
| CISD2 | 0.50784479 | 5.378622015 | 3.877213095 | 0.000417271 | 0.046257517 | 0.02081707 |
| SMARCD2 | -0.513373788 | 4.979282044 | -4.451105714 | 7.56E-05 | 0.026058233 | 1.543922435 |
| STX8 | -0.515372149 | 5.248922551 | -4.188343797 | 0.000166603 | 0.031778037 | 0.838615694 |
| ZBTB43 | -0.516028209 | 5.699546796 | -4.67440865 | 3.83E-05 | 0.024641026 | 2.151335453 |
| P2RY8 | -0.534939774 | 6.912731124 | -4.813619553 | 2.50E-05 | 0.023574257 | 2.532890118 |
| PRPF31 | -0.561409728 | 6.545995849 | -3.875278843 | 0.000419631 | 0.046257517 | 0.015802561 |
| SPATA13 | -0.567759852 | 6.833151422 | -4.408146445 | 8.61E-05 | 0.026058233 | 1.427843109 |
| RPL24 | -0.590532948 | 6.249800349 | -4.086938352 | 0.000225232 | 0.035733962 | 0.569772596 |
| SH3BGRL3 | -0.659216251 | 6.044540069 | -3.983754066 | 0.000305435 | 0.041174833 | 0.298427019 |
| RPS21 | -0.850919095 | 5.136310084 | -4.064396047 | 0.000240777 | 0.03749939 | 0.510295054 |

Supplementary Table 3. Functional enrichment analyses of DEGs.

|  | ID | Description | GeneRatio | BgRatio | pvalue | p.adjust | qvalue | geneID | Count |
| --- | --- | --- | --- | --- | --- | --- | --- | --- | --- |
| **BP** | GO:0019080 | viral gene expression | 4/47 | 191/18670 | 0.001342642 | 0.001342642 | 0.301579739 | JUN/EIF3A/RPL24/RPS21 | 4 |
| **BP** | GO:0035588 | G protein-coupled purinergic receptor signaling pathway | 2/47 | 24/18670 | 0.001652566 | 0.001652566 | 0.301579739 | P2RY11/P2RY8 | 2 |
| **BP** | GO:0036010 | protein localization to endosome | 2/47 | 24/18670 | 0.001652566 | 0.001652566 | 0.301579739 | RAB35/TMEM30A | 2 |
| **BP** | GO:0002181 | cytoplasmic translation | 3/47 | 100/18670 | 0.002037764 | 0.002037764 | 0.301579739 | EIF3A/RPL24/RPS21 | 3 |
| **BP** | GO:0000184 | nuclear-transcribed mRNA catabolic process, nonsense-mediated decay | 3/47 | 120/18670 | 0.003417144 | 0.003417144 | 0.301579739 | PARN/RPL24/RPS21 | 3 |
| **BP** | GO:0003014 | renal system process | 3/47 | 120/18670 | 0.003417144 | 0.003417144 | 0.301579739 | KLHL3/ADCY3/RAB11A | 3 |
| **BP** | GO:0003091 | renal water homeostasis | 2/47 | 36/18670 | 0.003700377 | 0.003700377 | 0.301579739 | ADCY3/RAB11A | 2 |
| **BP** | GO:0007190 | activation of adenylate cyclase activity | 2/47 | 38/18670 | 0.004115964 | 0.004115964 | 0.301579739 | ADCY3/P2RY11 | 2 |
| **BP** | GO:0007265 | Ras protein signal transduction | 5/47 | 448/18670 | 0.005210241 | 0.005210241 | 0.301579739 | RAB35/JUN/RAB11A/P2RY8/SPATA13 | 5 |
| **BP** | GO:0007409 | axonogenesis | 5/47 | 468/18670 | 0.006250592 | 0.006250592 | 0.301579739 | LLGL1/SEMA4C/RAB11A/PLCG1/RPL24 | 5 |
| **BP** | GO:0043628 | ncRNA 3'-end processing | 2/47 | 48/18670 | 0.006499601 | 0.006499601 | 0.301579739 | PARN/RPS21 | 2 |
| **BP** | GO:0033059 | cellular pigmentation | 2/47 | 50/18670 | 0.007036024 | 0.007036024 | 0.301579739 | TRAPPC6A/RAB11A | 2 |
| **BP** | GO:0010634 | positive regulation of epithelial cell migration | 3/47 | 171/18670 | 0.009112572 | 0.009112572 | 0.301579739 | JUN/RAB11A/PLCG1 | 3 |
| **BP** | GO:0090150 | establishment of protein localization to membrane | 4/47 | 332/18670 | 0.009602564 | 0.009602564 | 0.301579739 | TIMM10/RAB11A/RPL24/RPS21 | 4 |
| **BP** | GO:0019083 | viral transcription | 3/47 | 177/18670 | 0.010006597 | 0.010006597 | 0.301579739 | JUN/RPL24/RPS21 | 3 |
| **BP** | GO:0030104 | water homeostasis | 2/47 | 74/18670 | 0.014931929 | 0.014931929 | 0.301579739 | ADCY3/RAB11A | 2 |
| **BP** | GO:0048145 | regulation of fibroblast proliferation | 2/47 | 83/18670 | 0.018545806 | 0.018545806 | 0.301579739 | JUN/LTA | 2 |
| **BP** | GO:0048144 | fibroblast proliferation | 2/47 | 84/18670 | 0.01896802 | 0.01896802 | 0.301579739 | JUN/LTA | 2 |
| **BP** | GO:0009791 | post-embryonic development | 2/47 | 88/18670 | 0.02069718 | 0.02069718 | 0.301579739 | STK36/ATF5 | 2 |
| **BP** | GO:0043044 | ATP-dependent chromatin remodeling | 2/47 | 88/18670 | 0.02069718 | 0.02069718 | 0.301579739 | PTMA/SMARCD2 | 2 |
| **BP** | GO:1903076 | regulation of protein localization to plasma membrane | 2/47 | 95/18670 | 0.023874999 | 0.023874999 | 0.301579739 | RAB11A/STX8 | 2 |
| **BP** | GO:0030516 | regulation of axon extension | 2/47 | 96/18670 | 0.024344397 | 0.024344397 | 0.301579739 | SEMA4C/RAB11A | 2 |
| **BP** | GO:0098876 | vesicle-mediated transport to the plasma membrane | 2/47 | 96/18670 | 0.024344397 | 0.024344397 | 0.301579739 | LLGL1/RAB11A | 2 |
| **BP** | GO:0006171 | cAMP biosynthetic process | 1/47 | 10/18670 | 0.024896735 | 0.024896735 | 0.301579739 | ADCY3 | 1 |
| **BP** | GO:0021924 | cell proliferation in external granule layer | 1/47 | 10/18670 | 0.024896735 | 0.024896735 | 0.301579739 | ATF5 | 1 |
| **BP** | GO:0021930 | cerebellar granule cell precursor proliferation | 1/47 | 10/18670 | 0.024896735 | 0.024896735 | 0.301579739 | ATF5 | 1 |
| **BP** | GO:0042048 | olfactory behavior | 1/47 | 10/18670 | 0.024896735 | 0.024896735 | 0.301579739 | ADCY3 | 1 |
| **BP** | GO:0045657 | positive regulation of monocyte differentiation | 1/47 | 10/18670 | 0.024896735 | 0.024896735 | 0.301579739 | JUN | 1 |
| **BP** | GO:0046322 | negative regulation of fatty acid oxidation | 1/47 | 10/18670 | 0.024896735 | 0.024896735 | 0.301579739 | ACACB | 1 |
| **BP** | GO:0043473 | pigmentation | 2/47 | 98/18670 | 0.025294548 | 0.025294548 | 0.301579739 | TRAPPC6A/RAB11A | 2 |
| **BP** | GO:0019395 | fatty acid oxidation | 2/47 | 99/18670 | 0.025775258 | 0.025775258 | 0.301579739 | ACACB/CYP4V2 | 2 |
| **BP** | GO:1990542 | mitochondrial transmembrane transport | 2/47 | 101/18670 | 0.026747843 | 0.026747843 | 0.301579739 | ACACB/TIMM10 | 2 |
| **BP** | GO:0000478 | endonucleolytic cleavage involved in rRNA processing | 1/47 | 11/18670 | 0.027352783 | 0.027352783 | 0.301579739 | RPS21 | 1 |
| **BP** | GO:0000479 | endonucleolytic cleavage of tricistronic rRNA transcript (SSU-rRNA, 5.8S rRNA, LSU-rRNA) | 1/47 | 11/18670 | 0.027352783 | 0.027352783 | 0.301579739 | RPS21 | 1 |
| **BP** | GO:0002923 | regulation of humoral immune response mediated by circulating immunoglobulin | 1/47 | 11/18670 | 0.027352783 | 0.027352783 | 0.301579739 | LTA | 1 |
| **BP** | GO:0006853 | carnitine shuttle | 1/47 | 11/18670 | 0.027352783 | 0.027352783 | 0.301579739 | ACACB | 1 |
| **BP** | GO:0072321 | chaperone-mediated protein transport | 1/47 | 11/18670 | 0.027352783 | 0.027352783 | 0.301579739 | TIMM10 | 1 |
| **BP** | GO:0098887 | neurotransmitter receptor transport, endosome to postsynaptic membrane | 1/47 | 11/18670 | 0.027352783 | 0.027352783 | 0.301579739 | RAB11A | 1 |
| **BP** | GO:0099639 | neurotransmitter receptor transport, endosome to plasma membrane | 1/47 | 11/18670 | 0.027352783 | 0.027352783 | 0.301579739 | RAB11A | 1 |
| **BP** | GO:1904321 | response to forskolin | 1/47 | 11/18670 | 0.027352783 | 0.027352783 | 0.301579739 | ADCY3 | 1 |
| **BP** | GO:1904322 | cellular response to forskolin | 1/47 | 11/18670 | 0.027352783 | 0.027352783 | 0.301579739 | ADCY3 | 1 |
| **BP** | GO:0001667 | ameboidal-type cell migration | 4/47 | 461/18670 | 0.028391657 | 0.028391657 | 0.301579739 | JUN/SEMA4C/RAB11A/PLCG1 | 4 |
| **BP** | GO:0031330 | negative regulation of cellular catabolic process | 3/47 | 264/18670 | 0.028705966 | 0.028705966 | 0.301579739 | HIPK2/PARN/ACACB | 3 |
| **BP** | GO:0002863 | positive regulation of inflammatory response to antigenic stimulus | 1/47 | 12/18670 | 0.029802776 | 0.029802776 | 0.301579739 | LTA | 1 |
| **BP** | GO:0021889 | olfactory bulb interneuron differentiation | 1/47 | 12/18670 | 0.029802776 | 0.029802776 | 0.301579739 | ATF5 | 1 |
| **BP** | GO:0043922 | negative regulation by host of viral transcription | 1/47 | 12/18670 | 0.029802776 | 0.029802776 | 0.301579739 | JUN | 1 |
| **BP** | GO:0072017 | distal tubule development | 1/47 | 12/18670 | 0.029802776 | 0.029802776 | 0.301579739 | KLHL3 | 1 |
| **BP** | GO:0097201 | negative regulation of transcription from RNA polymerase II promoter in response to stress | 1/47 | 12/18670 | 0.029802776 | 0.029802776 | 0.301579739 | JUN | 1 |
| **BP** | GO:0099638 | endosome to plasma membrane protein transport | 1/47 | 12/18670 | 0.029802776 | 0.029802776 | 0.301579739 | RAB11A | 1 |
| **BP** | GO:0050769 | positive regulation of neurogenesis | 4/47 | 474/18670 | 0.031006346 | 0.031006346 | 0.301579739 | TMEM30A/LTA/RAB11A/MMD | 4 |
| **BP** | GO:0061387 | regulation of extent of cell growth | 2/47 | 110/18670 | 0.031304579 | 0.031304579 | 0.301579739 | SEMA4C/RAB11A | 2 |
| **BP** | GO:0021554 | optic nerve development | 1/47 | 13/18670 | 0.032246729 | 0.032246729 | 0.301579739 | RPL24 | 1 |
| **BP** | GO:0031953 | negative regulation of protein autophosphorylation | 1/47 | 13/18670 | 0.032246729 | 0.032246729 | 0.301579739 | JUN | 1 |
| **BP** | GO:0061029 | eyelid development in camera-type eye | 1/47 | 13/18670 | 0.032246729 | 0.032246729 | 0.301579739 | JUN | 1 |
| **BP** | GO:0061478 | response to platelet aggregation inhibitor | 1/47 | 13/18670 | 0.032246729 | 0.032246729 | 0.301579739 | ADCY3 | 1 |
| **BP** | GO:0001938 | positive regulation of endothelial cell proliferation | 2/47 | 112/18670 | 0.032356233 | 0.032356233 | 0.301579739 | JUN/PLCG1 | 2 |
| **BP** | GO:0051817 | modification of morphology or physiology of other organism involved in symbiotic interaction | 2/47 | 112/18670 | 0.032356233 | 0.032356233 | 0.301579739 | HIPK2/JUN | 2 |
| **BP** | GO:0022618 | ribonucleoprotein complex assembly | 3/47 | 277/18670 | 0.032439091 | 0.032439091 | 0.301579739 | EIF3A/PRPF31/RPL24 | 3 |
| **BP** | GO:1904375 | regulation of protein localization to cell periphery | 2/47 | 115/18670 | 0.03395963 | 0.03395963 | 0.301579739 | RAB11A/STX8 | 2 |
| **BP** | GO:0021535 | cell migration in hindbrain | 1/47 | 14/18670 | 0.034684656 | 0.034684656 | 0.301579739 | SEMA4C | 1 |
| **BP** | GO:0031667 | response to nutrient levels | 4/47 | 499/18670 | 0.036425974 | 0.036425974 | 0.301579739 | JUN/LTA/ACACB/P2RY11 | 4 |
| **BP** | GO:0071826 | ribonucleoprotein complex subunit organization | 3/47 | 291/18670 | 0.036731152 | 0.036731152 | 0.301579739 | EIF3A/PRPF31/RPL24 | 3 |
| **BP** | GO:0006098 | pentose-phosphate shunt | 1/47 | 15/18670 | 0.037116572 | 0.037116572 | 0.301579739 | RPIA | 1 |
| **BP** | GO:0006491 | N-glycan processing | 1/47 | 15/18670 | 0.037116572 | 0.037116572 | 0.301579739 | GANAB | 1 |
| **BP** | GO:0019321 | pentose metabolic process | 1/47 | 15/18670 | 0.037116572 | 0.037116572 | 0.301579739 | RPIA | 1 |
| **BP** | GO:0072599 | establishment of protein localization to endoplasmic reticulum | 2/47 | 122/18670 | 0.03781906 | 0.03781906 | 0.301579739 | RPL24/RPS21 | 2 |
| **BP** | GO:0051101 | regulation of DNA binding | 2/47 | 124/18670 | 0.038951437 | 0.038951437 | 0.301579739 | HIPK2/JUN | 2 |
| **BP** | GO:0030575 | nuclear body organization | 1/47 | 16/18670 | 0.039542491 | 0.039542491 | 0.301579739 | HIPK2 | 1 |
| **BP** | GO:0070863 | positive regulation of protein exit from endoplasmic reticulum | 1/47 | 16/18670 | 0.039542491 | 0.039542491 | 0.301579739 | TMEM30A | 1 |
| **BP** | GO:1902001 | fatty acid transmembrane transport | 1/47 | 16/18670 | 0.039542491 | 0.039542491 | 0.301579739 | ACACB | 1 |
| **BP** | GO:0006744 | ubiquinone biosynthetic process | 1/47 | 17/18670 | 0.041962428 | 0.041962428 | 0.301579739 | PDSS2 | 1 |
| **BP** | GO:0006978 | DNA damage response, signal transduction by p53 class mediator resulting in transcription of p21 class mediator | 1/47 | 17/18670 | 0.041962428 | 0.041962428 | 0.301579739 | HIPK2 | 1 |
| **BP** | GO:0048535 | lymph node development | 1/47 | 17/18670 | 0.041962428 | 0.041962428 | 0.301579739 | LTA | 1 |
| **BP** | GO:1901663 | quinone biosynthetic process | 1/47 | 17/18670 | 0.041962428 | 0.041962428 | 0.301579739 | PDSS2 | 1 |
| **BP** | GO:0009895 | negative regulation of catabolic process | 3/47 | 308/18670 | 0.04231788 | 0.04231788 | 0.301579739 | HIPK2/PARN/ACACB | 3 |
| **BP** | GO:0031998 | regulation of fatty acid beta-oxidation | 1/47 | 18/18670 | 0.044376397 | 0.044376397 | 0.301579739 | ACACB | 1 |
| **BP** | GO:0034199 | activation of protein kinase A activity | 1/47 | 18/18670 | 0.044376397 | 0.044376397 | 0.301579739 | ADCY3 | 1 |
| **BP** | GO:0035994 | response to muscle stretch | 1/47 | 18/18670 | 0.044376397 | 0.044376397 | 0.301579739 | JUN | 1 |
| **BP** | GO:0045056 | transcytosis | 1/47 | 18/18670 | 0.044376397 | 0.044376397 | 0.301579739 | RAB11A | 1 |
| **BP** | GO:0071318 | cellular response to ATP | 1/47 | 18/18670 | 0.044376397 | 0.044376397 | 0.301579739 | P2RY11 | 1 |
| **BP** | GO:0098877 | neurotransmitter receptor transport to plasma membrane | 1/47 | 18/18670 | 0.044376397 | 0.044376397 | 0.301579739 | RAB11A | 1 |
| **BP** | GO:0098969 | neurotransmitter receptor transport to postsynaptic membrane | 1/47 | 18/18670 | 0.044376397 | 0.044376397 | 0.301579739 | RAB11A | 1 |
| **BP** | GO:1904872 | regulation of telomerase RNA localization to Cajal body | 1/47 | 18/18670 | 0.044376397 | 0.044376397 | 0.301579739 | PARN | 1 |
| **BP** | GO:0046683 | response to organophosphorus | 2/47 | 134/18670 | 0.04480394 | 0.04480394 | 0.301579739 | JUN/P2RY11 | 2 |
| **BP** | GO:0002544 | chronic inflammatory response | 1/47 | 19/18670 | 0.046784413 | 0.046784413 | 0.301579739 | LTA | 1 |
| **BP** | GO:0002922 | positive regulation of humoral immune response | 1/47 | 19/18670 | 0.046784413 | 0.046784413 | 0.301579739 | LTA | 1 |
| **BP** | GO:0006337 | nucleosome disassembly | 1/47 | 19/18670 | 0.046784413 | 0.046784413 | 0.301579739 | SMARCD2 | 1 |
| **BP** | GO:0034138 | toll-like receptor 3 signaling pathway | 1/47 | 19/18670 | 0.046784413 | 0.046784413 | 0.301579739 | WDFY1 | 1 |
| **BP** | GO:0090685 | RNA localization to nucleus | 1/47 | 19/18670 | 0.046784413 | 0.046784413 | 0.301579739 | PARN | 1 |
| **BP** | GO:1990182 | exosomal secretion | 1/47 | 19/18670 | 0.046784413 | 0.046784413 | 0.301579739 | RAB11A | 1 |
| **BP** | GO:0016050 | vesicle organization | 3/47 | 325/18670 | 0.0483096 | 0.0483096 | 0.301579739 | TRAPPC6A/RAB11A/STX8 | 3 |
| **BP** | GO:0035023 | regulation of Rho protein signal transduction | 2/47 | 140/18670 | 0.048462552 | 0.048462552 | 0.301579739 | P2RY8/SPATA13 | 2 |
| **BP** | GO:0051188 | cofactor biosynthetic process | 3/47 | 326/18670 | 0.0486745 | 0.0486745 | 0.301579739 | ACACB/COX10/PDSS2 | 3 |
| **BP** | GO:0097734 | extracellular exosome biogenesis | 1/47 | 20/18670 | 0.049186489 | 0.049186489 | 0.301579739 | RAB11A | 1 |
| **CC** | GO:0030658 | transport vesicle membrane | 4/48 | 208/19717 | 0.001628343 | 0.001628343 | 0.058322469 | RAB35/TMEM30A/SEMA4C/TPRG1L | 4 |
| **CC** | GO:0005802 | trans-Golgi network | 4/48 | 236/19717 | 0.002576822 | 0.002576822 | 0.058322469 | LLGL1/TRAPPC6A/RAB11A/STX8 | 4 |
| **CC** | GO:0042788 | polysomal ribosome | 2/48 | 32/19717 | 0.002747422 | 0.002747422 | 0.058322469 | RPL24/RPS21 | 2 |
| **CC** | GO:0010008 | endosome membrane | 5/48 | 479/19717 | 0.006008795 | 0.006008795 | 0.10933296 | RAB35/LLGL1/RAB11A/MMD/STX8 | 5 |
| **CC** | GO:0001726 | ruffle | 3/48 | 172/19717 | 0.008462926 | 0.008462926 | 0.127505806 | SH3YL1/PLCG1/SPATA13 | 3 |
| **CC** | GO:0055037 | recycling endosome | 3/48 | 176/19717 | 0.009009708 | 0.009009708 | 0.127505806 | RAB35/RAB11A/STX8 | 3 |
| **CC** | GO:0005667 | transcription factor complex | 4/48 | 365/19717 | 0.011845794 | 0.011845794 | 0.136646538 | PBX2/HIPK2/JUN/ATF5 | 4 |
| **CC** | GO:0005844 | polysome | 2/48 | 72/19717 | 0.013309903 | 0.013309903 | 0.136646538 | RPL24/RPS21 | 2 |
| **CC** | GO:0098685 | Schaffer collateral - CA1 synapse | 2/48 | 82/19717 | 0.01702954 | 0.01702954 | 0.144601706 | RAB11A/PLCG1 | 2 |
| **CC** | GO:0032587 | ruffle membrane | 2/48 | 94/19717 | 0.022004602 | 0.022004602 | 0.172772523 | SH3YL1/SPATA13 | 2 |
| **CC** | GO:0005687 | U4 snRNP | 1/48 | 13/19717 | 0.031199005 | 0.031199005 | 0.172772523 | PRPF31 | 1 |
| **CC** | GO:0098993 | anchored component of synaptic vesicle membrane | 1/48 | 13/19717 | 0.031199005 | 0.031199005 | 0.172772523 | RAB35 | 1 |
| **CC** | GO:0005744 | TIM23 mitochondrial import inner membrane translocase complex | 1/48 | 14/19717 | 0.033559056 | 0.033559056 | 0.176438523 | TIMM10 | 1 |
| **CC** | GO:0016514 | SWI/SNF complex | 1/48 | 19/19717 | 0.045275129 | 0.045275129 | 0.180206927 | SMARCD2 | 1 |
| **MF** | GO:0033613 | activating transcription factor binding | 3/48 | 85/17697 | 0.001582474 | 0.001582474 | 0.150840613 | HIPK2/JUN/PTMA | 3 |
| **MF** | GO:0035586 | purinergic receptor activity | 2/48 | 25/17697 | 0.002076791 | 0.002076791 | 0.150840613 | P2RY11/P2RY8 | 2 |
| **MF** | GO:0016765 | transferase activity, transferring alkyl or aryl (other than methyl) groups | 2/48 | 58/17697 | 0.010810772 | 0.010810772 | 0.191348306 | COX10/PDSS2 | 2 |
| **MF** | GO:0030971 | receptor tyrosine kinase binding | 2/48 | 69/17697 | 0.015056205 | 0.015056205 | 0.191348306 | EIF3A/PLCG1 | 2 |
| **MF** | GO:0017022 | myosin binding | 2/48 | 71/17697 | 0.015893716 | 0.015893716 | 0.191348306 | LLGL1/RAB11A | 2 |
| **MF** | GO:0019905 | syntaxin binding | 2/48 | 75/17697 | 0.017627339 | 0.017627339 | 0.191348306 | RAB11A/STX8 | 2 |
| **MF** | GO:0046332 | SMAD binding | 2/48 | 80/17697 | 0.019901915 | 0.019901915 | 0.191348306 | HIPK2/JUN | 2 |
| **MF** | GO:0004016 | adenylate cyclase activity | 1/48 | 10/17697 | 0.026801306 | 0.026801306 | 0.191348306 | ADCY3 | 1 |
| **MF** | GO:0005165 | neurotrophin receptor binding | 1/48 | 10/17697 | 0.026801306 | 0.026801306 | 0.191348306 | PLCG1 | 1 |
| **MF** | GO:0019869 | chloride channel inhibitor activity | 1/48 | 10/17697 | 0.026801306 | 0.026801306 | 0.191348306 | STX8 | 1 |
| **MF** | GO:0046790 | virion binding | 1/48 | 10/17697 | 0.026801306 | 0.026801306 | 0.191348306 | HIPK2 | 1 |
| **MF** | GO:0005543 | phospholipid binding | 4/48 | 427/17697 | 0.028233104 | 0.028233104 | 0.191348306 | MFGE8/RAB35/SH3YL1/WDFY1 | 4 |
| **MF** | GO:0030621 | U4 snRNA binding | 1/48 | 11/17697 | 0.029442428 | 0.029442428 | 0.191348306 | PRPF31 | 1 |
| **MF** | GO:0000980 | RNA polymerase II distal enhancer sequence-specific DNA binding | 2/48 | 99/17697 | 0.029576714 | 0.029576714 | 0.191348306 | JUN/SMARCD2 | 2 |
| **MF** | GO:0004535 | poly(A)-specific ribonuclease activity | 1/48 | 13/17697 | 0.034703636 | 0.034703636 | 0.191348306 | PARN | 1 |
| **MF** | GO:0015926 | glucosidase activity | 1/48 | 14/17697 | 0.037323757 | 0.037323757 | 0.191348306 | GANAB | 1 |
| **MF** | GO:0035497 | cAMP response element binding | 1/48 | 14/17697 | 0.037323757 | 0.037323757 | 0.191348306 | JUN | 1 |
| **MF** | GO:0009055 | electron transfer activity | 2/48 | 114/17697 | 0.038281117 | 0.038281117 | 0.191348306 | COX10/SH3BGRL3 | 2 |
| **MF** | GO:0071837 | HMG box domain binding | 1/48 | 16/17697 | 0.042543126 | 0.042543126 | 0.191348306 | JUN | 1 |
| **MF** | GO:0017081 | chloride channel regulator activity | 1/48 | 17/17697 | 0.045142409 | 0.045142409 | 0.191348306 | STX8 | 1 |
| **MF** | GO:0030676 | Rac guanyl-nucleotide exchange factor activity | 1/48 | 17/17697 | 0.045142409 | 0.045142409 | 0.191348306 | SPATA13 | 1 |
| **MF** | GO:0038191 | neuropilin binding | 1/48 | 18/17697 | 0.047734783 | 0.047734783 | 0.191348306 | SEMA4C | 1 |

Supplementary Table 4. KEGG analysis.

| ID | Description | GeneRatio | BgRatio | pvalue | p.adjust | qvalue | geneID | Count |
| --- | --- | --- | --- | --- | --- | --- | --- | --- |
| hsa05171 | Coronavirus disease - COVID-19 | 4月26日 | 232/8108 | 0.005950154 | 0.005950154 | 0.344851829 | 3725/5335/6152/6227 | 4 |
| hsa04962 | Vasopressin-regulated water reabsorption | 2月26日 | 44/8108 | 0.008612072 | 0.008612072 | 0.344851829 | 109/8766 | 2 |
| hsa05110 | Vibrio cholerae infection | 2月26日 | 50/8108 | 0.011021497 | 0.011021497 | 0.344851829 | 109/5335 | 2 |
| hsa05120 | Epithelial cell signaling in Helicobacter pylori infection | 2月26日 | 70/8108 | 0.020893841 | 0.020893841 | 0.344851829 | 3725/5335 | 2 |
| hsa04012 | ErbB signaling pathway | 2月26日 | 85/8108 | 0.029995393 | 0.029995393 | 0.344851829 | 3725/5335 | 2 |
| hsa05166 | Human T-cell leukemia virus 1 infection | 3月26日 | 219/8108 | 0.031989803 | 0.031989803 | 0.344851829 | 3725/4049/109 | 3 |
| hsa05235 | PD-L1 expression and PD-1 checkpoint pathway in cancer | 2月26日 | 89/8108 | 0.032647051 | 0.032647051 | 0.344851829 | 3725/5335 | 2 |
| hsa04658 | Th1 and Th2 cell differentiation | 2月26日 | 92/8108 | 0.034694707 | 0.034694707 | 0.344851829 | 3725/5335 | 2 |
| hsa04912 | GnRH signaling pathway | 2月26日 | 93/8108 | 0.035388269 | 0.035388269 | 0.344851829 | 3725/109 | 2 |
| hsa04714 | Thermogenesis | 3月26日 | 232/8108 | 0.037034201 | 0.037034201 | 0.344851829 | 109/1352/6603 | 3 |
| hsa01522 | Endocrine resistance | 2月26日 | 98/8108 | 0.038936891 | 0.038936891 | 0.344851829 | 3725/109 | 2 |
| hsa04750 | Inflammatory mediator regulation of TRP channels | 2月26日 | 98/8108 | 0.038936891 | 0.038936891 | 0.344851829 | 109/5335 | 2 |
| hsa05231 | Choline metabolism in cancer | 2月26日 | 98/8108 | 0.038936891 | 0.038936891 | 0.344851829 | 3725/5335 | 2 |
| hsa04933 | AGE-RAGE signaling pathway in diabetic complications | 2月26日 | 100/8108 | 0.040393331 | 0.040393331 | 0.344851829 | 3725/5335 | 2 |
| hsa04972 | Pancreatic secretion | 2月26日 | 102/8108 | 0.041870449 | 0.041870449 | 0.344851829 | 109/8766 | 2 |
| hsa04064 | NF-kappa B signaling pathway | 2月26日 | 104/8108 | 0.043367944 | 0.043367944 | 0.344851829 | 4049/5335 | 2 |
| hsa04660 | T cell receptor signaling pathway | 2月26日 | 104/8108 | 0.043367944 | 0.043367944 | 0.344851829 | 3725/5335 | 2 |
| hsa04659 | Th17 cell differentiation | 2月26日 | 107/8108 | 0.045651739 | 0.045651739 | 0.344851829 | 3725/5335 | 2 |
| hsa04668 | Th17 cell differentiation | 2月26日 | 112/8108 | 0.049555749 | 0.049555749 | 0.344851829 | 3725/4049 | 2 |

Supplementary Table 5. The enrichment results of hallmark and immunologic signature function terms for each diagnostic marker.

| NAME | SIZE | ES | NES | NOM p-val | FDR q-val | FWER p-val | RANK AT MAX | LEADING EDGE |
| --- | --- | --- | --- | --- | --- | --- | --- | --- |
| HALLMARK_CHOLESTEROL_HOMEOSTASIS | 72 | 0.44037542 | 1.7552236 | 0.001941748 | 0.10351001 | 0.108 | 1828 | tags=28%, list=11%, signal=31% |
| **Immunologic signature gene set** |  |  |  |  |  |  |  |  |
| NAME | SIZE | ES | NES | NOM p-val | FDR q-val | FWER p-val | RANK AT MAX | LEADING EDGE |
| FRANCO_BLOOD_SANOFI_PASTEUR_SA_INACTIVATED_INFLUENZA_VACCINE_CORRELATED_WITH_ANTIBODY_RESPONSE_AGE_18_40YO_1DY_NEGATIVE | 18 | 0.7796379 | 1.99456 | 0 | 0.20921907 | 0.077 | 2246 | tags=67%, list=13%, signal=77% |
| CTRL_VS_B_ABORTUS_4H_MAC_CELL_LINE_UP | 178 | 0.37242377 | 1.6903436 | 0 | 1 | 0.762 | 1712 | tags=22%, list=10%, signal=25% |
| UNTREATED_VS_AD5_T425A_HEXON_INF_MOUSE_LUNG_DC_UP | 176 | 0.33953393 | 1.6370347 | 0 | 1 | 0.847 | 2534 | tags=23%, list=15%, signal=26% |
| SUBCAPSULAR_CORTICAL_REGION_VS_WHOLE_CORTEX_THYMUS_UP | 186 | 0.3331857 | 1.5692834 | 0 | 1 | 0.955 | 2101 | tags=23%, list=12%, signal=25% |
| CTRL_VS_FOXP3_OVEREXPR_TCONV_DN | 167 | 0.32306838 | 1.5641217 | 0 | 1 | 0.958 | 2289 | tags=23%, list=13%, signal=26% |
| CTRL_VS_LPS_4H_BMDC_DN | 178 | 0.30828035 | 1.5448738 | 0 | 1 | 0.972 | 2947 | tags=25%, list=17%, signal=30% |
| 0.5H_VS_4H_PAM3CSK4_BMDC_DN | 178 | 0.33536503 | 1.5413283 | 0.002008032 | 1 | 0.974 | 2454 | tags=26%, list=14%, signal=31% |
| PRIMARY_VS_SECONDARY_ACUTE_LCMV_INF_CD8_TCELL_DN | 170 | 0.3184654 | 1.5559716 | 0.002087683 | 1 | 0.964 | 2338 | tags=24%, list=14%, signal=28% |
| RETINOIC_ACID_VS_RETINOIC_ACID_AND_PAM2CSK4_STIM_FOLLICULAR_DC_UP | 177 | 0.31939024 | 1.585626 | 0.00212766 | 1 | 0.937 | 2685 | tags=27%, list=16%, signal=32% |
| RARA_AGONIST_AM580_VS_AM580_AND_ROSIGLITAZONE_TREATED_DC_UP | 177 | 0.34168428 | 1.5530096 | 0.004338395 | 1 | 0.966 | 2530 | tags=25%, list=15%, signal=30% |
| 33D1_POS_DC_VS_BCELL_UP | 144 | 0.33791 | 1.5452199 | 0.004484305 | 1 | 0.972 | 2687 | tags=26%, list=16%, signal=30% |
| CTRL_VS_HCMV_INF_MONOCYTES_DN | 150 | 0.3384613 | 1.6334462 | 0.006 | 1 | 0.853 | 1459 | tags=17%, list=9%, signal=18% |
| 0.5H_VS_4H_LPS_BMDC_DN | 178 | 0.32616174 | 1.5663291 | 0.008196721 | 1 | 0.957 | 2908 | tags=26%, list=17%, signal=31% |
| HOFT_CD4_POSITIVE_ALPHA_BETA_MEMORY_T_CELL_BCG_VACCINE_AGE_18_45YO_56D_TOP_100_DEG_AFTER_IN_VITRO_RE_STIMULATION_UP | 16 | 0.7353726 | 1.8431892 | 0.008385744 | 1 | 0.337 | 1290 | tags=50%, list=8%, signal=54% |
| ANDERSON_BLOOD_CN54GP140_ADJUVANTED_WITH_GLA_AF_AGE_18_45YO_1DY_UP | 73 | 0.46107915 | 1.6781307 | 0.014705882 | 1 | 0.786 | 3146 | tags=38%, list=19%, signal=47% |
| ERWIN_COHEN_PBMC_TC_83_AGE_18_45YO_NON_RESPONDERS_PREVIOUSLY_IMMUNIZED_24HR_DEG_CANONICAL_PATHWAY_MEMBERS_UP | 18 | 0.6051542 | 1.643238 | 0.021868788 | 1 | 0.838 | 3131 | tags=50%, list=18%, signal=61% |
| ANDERSON_BLOOD_CN54GP140_ADJUVANTED_WITH_GLA_AF_AGE_18_45YO_7DY_UP | 30 | 0.584812 | 1.6827013 | 0.02536998 | 1 | 0.777 | 3058 | tags=53%, list=18%, signal=65% |
| NAIVE_VS_IL2RALOW_DAY3_EFF_CD8_TCELL_DN | 164 | 0.38896215 | 1.5451313 | 0.034557234 | 1 | 0.972 | 1828 | tags=29%, list=11%, signal=32% |
| THAKAR_PBMC_INACTIVATED_INFLUENZA_AGE_70PLS_NONRESPONDER_2DY_UP | 93 | 0.61833215 | 1.6946479 | 0.043564357 | 1 | 0.749 | 3078 | tags=56%, list=18%, signal=68% |
| THYMIC_DC_VS_THYMIC_MACROPHAGE_DN | 177 | -0.34891602 | -1.550948 | 0.001901141 | 1 | 0.97 | 2107 | tags=26%, list=12%, signal=29% |
| 3H_VS_24H_POLYIC_STIM_DC_UP | 143 | -0.43418896 | -1.7494988 | 0.00204918 | 1 | 0.607 | 1902 | tags=28%, list=11%, signal=31% |
| WT_VS_KLF3_KO_BCELL_DN | 181 | -0.40125218 | -1.685572 | 0.003875969 | 1 | 0.775 | 2991 | tags=31%, list=18%, signal=37% |
| WT_VS_KLF2_KO_LPS_STIM_MACROPHAGE_UP | 181 | -0.35588032 | -1.5662568 | 0.004040404 | 1 | 0.957 | 3680 | tags=34%, list=22%, signal=43% |
| 0H_VS_1H_IN_VITRO_ACT_CD4_TCELL_UP | 151 | -0.37546277 | -1.5837345 | 0.007648184 | 1 | 0.937 | 1981 | tags=27%, list=12%, signal=30% |
| LOW_DOSE_B_MALAYI_VS_M_TUBERCULOSIS_MAC_UP | 185 | -0.41934615 | -1.7611058 | 0.007736944 | 1 | 0.573 | 2248 | tags=31%, list=13%, signal=35% |
| CTRL_VS_IGE_STIM_MAST_CELL_DN | 175 | -0.37655973 | -1.610422 | 0.010162601 | 1 | 0.908 | 2071 | tags=27%, list=12%, signal=30% |
| PPARG_LIGAND_ROSIGLITAZONE_VS_RARA_AGONIST_AM580_TREATED_DC_DN | 180 | -0.35817438 | -1.5491799 | 0.011741683 | 1 | 0.971 | 1537 | tags=22%, list=9%, signal=24% |
| CTRL_VS_BORRELIA_BIRGDOFERI_INF_ENDOTHELIAL_CELL_DN | 136 | -0.3891061 | -1.5841986 | 0.012605042 | 1 | 0.937 | 4257 | tags=39%, list=25%, signal=52% |
| CTRL_VS_A3R_ACTIVATION_MAST_CELL_UP | 175 | -0.33689845 | -1.5174116 | 0.013806706 | 1 | 0.986 | 2087 | tags=24%, list=12%, signal=27% |
| CTRL_VS_RETINOIC_ACID_TREATED_CD4_TCELL_DN | 176 | -0.33528763 | -1.5116717 | 0.014285714 | 1 | 0.988 | 3638 | tags=30%, list=21%, signal=38% |
| LPS_VS_LPS_AND_IL10_STIM_MACROPHAGE_45MIN_DN | 171 | -0.38206664 | -1.5729434 | 0.016194332 | 1 | 0.951 | 2794 | tags=29%, list=16%, signal=35% |
| CTRL_VS_M_TUBERCULOSIS_MAC_UP | 178 | -0.393246 | -1.6021522 | 0.017175572 | 1 | 0.914 | 2418 | tags=31%, list=14%, signal=36% |
| PATEL_SKIN_OF_BODY_ZOSTAVAX_AGE_70_93YO_VZV_CHALLENGE_3DY_DN | 29 | -0.46663174 | -1.5309569 | 0.026859503 | 1 | 0.982 | 2642 | tags=31%, list=16%, signal=37% |
| DC_VS_MAC_DN | 181 | -0.38043758 | -1.553831 | 0.02851711 | 1 | 0.966 | 2843 | tags=32%, list=17%, signal=38% |
| **LTA** |  |  |  |  |  |  |  |  |
| **Hallmark gene set** |  |  |  |  |  |  |  |  |
| NAME | SIZE | ES | NES | NOM p-val | FDR q-val | FWER p-val | RANK AT MAX | LEADING EDGE |
| HALLMARK_CHOLESTEROL_HOMEOSTASIS | 72 | 0.3676902 | 1.4530039 | 0.041666668 | 0.73104304 | 0.542 | 1798 | tags=24%, list=11%, signal=26% |
| HALLMARK_TGF_BETA_SIGNALING | 54 | -0.47019663 | -1.6186008 | 0.012195122 | 0.754073 | 0.272 | 2225 | tags=35%, list=13%, signal=40% |
| HALLMARK_XENOBIOTIC_METABOLISM | 197 | -0.37679464 | -1.5475031 | 0.022633744 | 0.60682243 | 0.375 | 3076 | tags=31%, list=18%, signal=37% |
| HALLMARK_ADIPOGENESIS | 185 | -0.3716257 | -1.4992487 | 0.0331384 | 0.427108 | 0.454 | 3490 | tags=38%, list=21%, signal=48% |
| HALLMARK_ANGIOGENESIS | 36 | -0.48386273 | -1.5457112 | 0.036809817 | 0.40878746 | 0.378 | 3888 | tags=36%, list=23%, signal=47% |
| **Immunologic signature gene set** |  |  |  |  |  |  |  |  |
| NAME | SIZE | ES | NES | NOM p-val | FDR q-val | FWER p-val | RANK AT MAX | LEADING EDGE |
| NOD2_TRANSD_VS_CTRL_TRANSD_HEK293_MDP_STIM_2H_UP | 170 | 0.3422575 | 1.519707 | 0.001984127 | 1 | 0.975 | 3678 | tags=31%, list=22%, signal=39% |
| CTRL_VS_FOXP3_OVEREXPR_TCONV_DN | 167 | 0.30702633 | 1.4737276 | 0.003875969 | 1 | 0.991 | 2361 | tags=24%, list=14%, signal=28% |
| UNSTIM_VS_6H_MDP_STIM_NOD2_TRANSDUCED_HEK293T_CELL_UP | 177 | 0.3031818 | 1.4606068 | 0.004106776 | 1 | 0.993 | 3131 | tags=24%, list=18%, signal=29% |
| CTRL_VS_LPS_4H_BMDC_DN | 178 | 0.30089983 | 1.4737368 | 0.004115226 | 1 | 0.991 | 3721 | tags=31%, list=22%, signal=40% |
| ANDERSON_BLOOD_CN54GP140_ADJUVANTED_WITH_GLA_AF_AGE_18_45YO_7DY_UP | 30 | 0.6223433 | 1.7629776 | 0.004237288 | 1 | 0.562 | 2736 | tags=63%, list=16%, signal=75% |
| SOCS3_KO_VS_IFNG_KO_LIVER_UP | 165 | 0.3001005 | 1.4439605 | 0.00589391 | 1 | 0.994 | 2897 | tags=27%, list=17%, signal=32% |
| FRANCO_BLOOD_SANOFI_PASTEUR_SA_INACTIVATED_INFLUENZA_VACCINE_CORRELATED_WITH_ANTIBODY_RESPONSE_AGE_18_40YO_1DY_NEGATIVE | 18 | 0.7298898 | 1.817455 | 0.007889546 | 1 | 0.412 | 2214 | tags=72%, list=13%, signal=83% |
| ANDERSON_BLOOD_CN54GP140_ADJUVANTED_WITH_GLA_AF_AGE_18_45YO_1DY_UP | 73 | 0.46665928 | 1.7011065 | 0.012024048 | 1 | 0.734 | 3364 | tags=45%, list=20%, signal=56% |
| HEALTHY_VS_MCMV_INFECTION_CD11B_DC_IFNAR_KO_UP | 164 | 0.41231865 | 1.6746094 | 0.013513514 | 1 | 0.793 | 3012 | tags=34%, list=18%, signal=40% |
| CTRL_VS_B_ABORTUS_4H_MAC_CELL_LINE_UP | 178 | 0.33059663 | 1.4910111 | 0.013645224 | 1 | 0.988 | 1903 | tags=24%, list=11%, signal=27% |
| TREG_VS_TCONV_DN | 153 | 0.3216671 | 1.4811742 | 0.013888889 | 1 | 0.988 | 3049 | tags=26%, list=18%, signal=32% |
| MED_VS_SCS_MAC_LN_DN | 173 | 0.40207365 | 1.6607256 | 0.015503876 | 1 | 0.821 | 2999 | tags=35%, list=18%, signal=42% |
| WT_VS_FOXO1_FOXO3_KO_TREG_DN | 161 | 0.37459853 | 1.5586897 | 0.01996008 | 1 | 0.952 | 3671 | tags=35%, list=22%, signal=45% |
| IL4_AND_ANTI_IL12_VS_UNTREATED_2H_ACT_CD4_TCELL_UP | 148 | 0.33553964 | 1.4404526 | 0.02631579 | 1 | 0.995 | 2994 | tags=30%, list=18%, signal=37% |
| HOFT_CD4_POSITIVE_ALPHA_BETA_MEMORY_T_CELL_BCG_VACCINE_AGE_18_45YO_56D_TOP_100_DEG_AFTER_IN_VITRO_RE_STIMULATION_UP | 16 | 0.6534125 | 1.5822661 | 0.029473685 | 1 | 0.936 | 2742 | tags=56%, list=16%, signal=67% |
| WT_VS_IFNAR_KO_CD11B_DC_UP | 164 | 0.40253 | 1.5484906 | 0.03773585 | 1 | 0.958 | 3006 | tags=37%, list=18%, signal=45% |
| LPS_VS_LPS_AND_IL10_STIM_MACROPHAGE_45MIN_DN | 171 | -0.4626896 | -1.943305 | 0 | 0.98270303 | 0.162 | 2257 | tags=32%, list=13%, signal=37% |
| CTRL_VS_L_MAJOR_DC_UP | 186 | -0.41858187 | -1.7758967 | 0 | 1 | 0.545 | 1963 | tags=29%, list=12%, signal=32% |
| DC_VS_MAC_DN | 181 | -0.42788127 | -1.7792064 | 0.00189394 | 1 | 0.537 | 2995 | tags=38%, list=18%, signal=46% |
| CTRL_VS_L_DONOVANI_MAC_UP | 183 | -0.5230449 | -1.8621625 | 0.001923077 | 1 | 0.344 | 2698 | tags=50%, list=16%, signal=58% |
| CTRL_VS_M_TUBERCULOSIS_MAC_UP | 178 | -0.4257678 | -1.7277933 | 0.003883495 | 1 | 0.66 | 3398 | tags=43%, list=20%, signal=54% |
| 0.5H_VS_24H_POLYIC_BMDC_UP | 181 | -0.4313295 | -1.7728063 | 0.005524862 | 1 | 0.549 | 3412 | tags=34%, list=20%, signal=42% |
| 3H_VS_24H_POLYIC_STIM_DC_UP | 143 | -0.4165011 | -1.7251936 | 0.005825243 | 0.99207693 | 0.666 | 1619 | tags=27%, list=10%, signal=30% |
| 1H_VS_60H_ACT_CD4_TCELL_WITH_TGFB_IL6_UP | 183 | -0.45220235 | -1.7402449 | 0.00589391 | 1 | 0.626 | 2761 | tags=39%, list=16%, signal=46% |
| LOW_DOSE_B_MALAYI_VS_M_TUBERCULOSIS_MAC_UP | 185 | -0.4054106 | -1.709359 | 0.005940594 | 0.993783 | 0.716 | 2548 | tags=32%, list=15%, signal=38% |
| YOUNG_VS_OLD_DONOR_MEMORY_CD4_TCELL_40H_TSST_ACT_DN | 177 | -0.50952387 | -1.7427614 | 0.005988024 | 1 | 0.619 | 1975 | tags=41%, list=12%, signal=46% |
| UNTREATED_VS_PIOGLIZATONE_TREATED_CD4_TCELL_PPARG1_AND_FOXP3_TRASDUCED_DN | 186 | -0.4832042 | -1.772149 | 0.006072875 | 1 | 0.55 | 2192 | tags=37%, list=13%, signal=42% |
| 4H_VS_16H_IFNG_IN_CD8POS_DC_DN | 181 | -0.49452862 | -1.7260014 | 0.00617284 | 1 | 0.664 | 1937 | tags=32%, list=11%, signal=36% |
| BCELL_VS_BASOPHIL_DN | 176 | -0.4441267 | -1.7352558 | 0.00631579 | 1 | 0.635 | 3066 | tags=39%, list=18%, signal=47% |
| EFFECTOR_VS_EXHAUSTED_CD8_TCELL_UP | 176 | -0.49414587 | -1.7761453 | 0.007662835 | 1 | 0.545 | 2725 | tags=41%, list=16%, signal=49% |
| LI_PBMC_MENOMUNE_A_C_Y_W_135_AGE_18_45YO_CORRELATED_WITH_ANTIBODY_RESPONSE_3DY_POSITIVE | 43 | -0.6761401 | -1.8166282 | 0.008403362 | 1 | 0.444 | 2163 | tags=53%, list=13%, signal=61% |
| SCHERER_PBMC_APSV_WETVAX_AGE_18_32YO_2_TO_4DY_DN | 32 | -0.60614526 | -1.7191644 | 0.009689922 | 0.94105357 | 0.685 | 1674 | tags=41%, list=10%, signal=45% |
| NAIVE_VS_IGM_MEMORY_BCELL_DN | 175 | -0.44135073 | -1.6974509 | 0.01004016 | 1 | 0.734 | 2478 | tags=35%, list=15%, signal=40% |
| MAST_CELL_VS_CENT_MEMORY_CD4_TCELL_UP | 182 | -0.49556845 | -1.7192638 | 0.011583012 | 0.9954398 | 0.684 | 4145 | tags=52%, list=24%, signal=68% |
| HOFT_CD4_POSITIVE_ALPHA_BETA_MEMORY_T_CELL_BCG_VACCINE_AGE_18_45YO_56D_TOP_100_DEG_AFTER_IN_VITRO_RE_STIMULATION_DN | 42 | -0.5439751 | -1.7549165 | 0.013888889 | 1 | 0.596 | 4939 | tags=57%, list=29%, signal=80% |
| LI_PBMC_MENACTRA_AGE_18_45YO_CORRELATED_WITH_ANTI_DT_ANTIBODY_3DY_NEGATIVE | 27 | -0.66911703 | -1.7397386 | 0.014861995 | 1 | 0.626 | 3198 | tags=67%, list=19%, signal=82% |
| **FAM179B** |  |  |  |  |  |  |  |  |
| **Hallmark gene set** |  |  |  |  |  |  |  |  |
| NAME | SIZE | ES | NES | NOM p-val | FDR q-val | FWER p-val | RANK AT MAX | LEADING EDGE |
| HALLMARK_CHOLESTEROL_HOMEOSTASIS | 72 | 0.3961792 | 1.5559564 | 0.020491803 | 0.37384745 | 0.38 | 1681 | tags=25%, list=10%, signal=28% |
| HALLMARK_UV_RESPONSE_UP | 149 | -0.3454894 | -1.4765813 | 0.04238921 | 0.9787928 | 0.529 | 2645 | tags=30%, list=16%, signal=35% |
| **Immunologic signature gene set** |  |  |  |  |  |  |  |  |
| NAME | SIZE | ES | NES | NOM p-val | FDR q-val | FWER p-val | RANK AT MAX | LEADING EDGE |
| PRIMARY_VS_SECONDARY_MEMORY_CD8_TCELL_UP | 170 | 0.33420363 | 1.6017388 | 0 | 1 | 0.929 | 2440 | tags=21%, list=14%, signal=24% |
| CTRL_VS_FOXP3_OVEREXPR_TCONV_DN | 167 | 0.31898996 | 1.5043193 | 0.002293578 | 1 | 0.986 | 2970 | tags=29%, list=17%, signal=34% |
| FRANCO_BLOOD_SANOFI_PASTEUR_SA_INACTIVATED_INFLUENZA_VACCINE_CORRELATED_WITH_ANTIBODY_RESPONSE_AGE_18_40YO_1DY_NEGATIVE | 18 | 0.72365457 | 1.7812147 | 0.004106776 | 1 | 0.528 | 838 | tags=50%, list=5%, signal=53% |
| ARTHRITIC_SYNOVIAL_FLUID_VS_HEALTHY_MACROPHAGE_DN | 130 | 0.35787126 | 1.557992 | 0.006024096 | 1 | 0.962 | 2649 | tags=27%, list=16%, signal=32% |
| CTRL_VS_B_ABORTUS_4H_MAC_CELL_LINE_UP | 178 | 0.35164687 | 1.5835923 | 0.006122449 | 1 | 0.944 | 1868 | tags=25%, list=11%, signal=28% |
| ANDERSON_BLOOD_CN54GP140_ADJUVANTED_WITH_GLA_AF_AGE_18_45YO_1DY_UP | 73 | 0.4885671 | 1.7717874 | 0.007984032 | 1 | 0.548 | 3847 | tags=49%, list=23%, signal=63% |
| CTRL_VS_WEST_EQUINE_ENC_VIRUS_MATURE_NEURON_CELL_LINE_UP | 170 | 0.30772573 | 1.4474883 | 0.008064516 | 1 | 0.997 | 2922 | tags=27%, list=17%, signal=32% |
| PRIMARY_VS_SECONDARY_ACUTE_LCMV_INF_CD8_TCELL_DN | 170 | 0.307753 | 1.476555 | 0.008130081 | 1 | 0.991 | 1778 | tags=20%, list=10%, signal=22% |
| THAKAR_PBMC_INACTIVATED_INFLUENZA_AGE_70PLS_NONRESPONDER_2DY_UP | 93 | 0.67605203 | 1.793011 | 0.009803922 | 1 | 0.491 | 2365 | tags=56%, list=14%, signal=65% |
| UNSTIM_VS_LPS_AND_IL10_STIM_IL10_KO_NFKBP50_KO_MACROPHAGE_UP | 157 | 0.33132946 | 1.4896125 | 0.010548524 | 1 | 0.988 | 2669 | tags=25%, list=16%, signal=29% |
| UNTREATED_VS_PIOGLITAZONE_TREATED_CD4_TCELL_PPARG1_AND_FOXP3_TRASDUCED_UP | 166 | 0.330419 | 1.4893355 | 0.014141414 | 1 | 0.988 | 3006 | tags=30%, list=18%, signal=36% |
| ANDERSON_BLOOD_CN54GP140_ADJUVANTED_WITH_GLA_AF_AGE_18_45YO_7DY_UP | 30 | 0.60551 | 1.7108232 | 0.014256619 | 1 | 0.714 | 3438 | tags=67%, list=20%, signal=83% |
| WT_VS_HDAC7_KO_DOUBLE_POSITIVE_THYMOCYTE_UP | 155 | 0.32561842 | 1.4448812 | 0.014522822 | 1 | 0.997 | 2532 | tags=26%, list=15%, signal=31% |
| PRE_VS_DAY7_FLU_VACCINE_BCELL_DN | 171 | 0.32518885 | 1.449684 | 0.017716536 | 1 | 0.996 | 2789 | tags=29%, list=16%, signal=34% |
| HEALTHY_VS_LUPUS_RESTING_CD4_TCELL_DN | 107 | 0.34053987 | 1.5107483 | 0.01953125 | 1 | 0.984 | 1799 | tags=20%, list=11%, signal=22% |
| C57BL6_VS_NOD_FOXP3_IRES_GFP_TCONV_DN | 145 | 0.31329408 | 1.4212211 | 0.025052192 | 1 | 0.999 | 2208 | tags=23%, list=13%, signal=27% |
| HOFT_CD4_POSITIVE_ALPHA_BETA_MEMORY_T_CELL_BCG_VACCINE_AGE_18_45YO_56D_TOP_100_DEG_AFTER_IN_VITRO_RE_STIMULATION_UP | 16 | 0.6782138 | 1.644431 | 0.026052104 | 1 | 0.858 | 3960 | tags=88%, list=23%, signal=114% |
| EX_VIVO_VS_DEC205_CONVERSION_DN | 173 | 0.3390535 | 1.4573556 | 0.027944112 | 1 | 0.993 | 2486 | tags=27%, list=15%, signal=32% |
| LSK_VS_NKCELL_UP | 164 | 0.3585565 | 1.5025375 | 0.028301887 | 1 | 0.987 | 2052 | tags=24%, list=12%, signal=27% |
| CTRL_VS_ANTI_IGM_STIM_ZFX_KO_BCELL_12H_UP | 130 | 0.34222007 | 1.4760816 | 0.030364372 | 1 | 0.991 | 2585 | tags=26%, list=15%, signal=31% |
| MATSUMIYA_PBMC_MODIFIED_VACCINIA_ANKARA_VACCINE_AGE_18_55YO_VACCINATED_VS_CONTROL_TREATED_IN_VITRO_WITH_WILD_TYPE_MVA_6HR_UP | 23 | 0.48238295 | 1.5399387 | 0.035789475 | 1 | 0.967 | 2585 | tags=39%, list=15%, signal=46% |
| HEALTHY_VS_MCMV_INFECTION_CD11B_DC_IFNAR_KO_UP | 164 | 0.39536318 | 1.5719377 | 0.036072146 | 1 | 0.948 | 3402 | tags=38%, list=20%, signal=47% |
| WT_VS_DICER_KO_TREG_DN | 139 | 0.3745513 | 1.4655621 | 0.03696498 | 1 | 0.992 | 2683 | tags=31%, list=16%, signal=36% |
| UNSTIM_VS_MCSF_TREATED_MONOCYTE_DAY3_UP | 175 | 0.3612636 | 1.429127 | 0.04233871 | 1 | 0.998 | 1955 | tags=27%, list=12%, signal=31% |
| DC_VS_MAC_DN | 181 | -0.4505076 | -1.8256218 | 0 | 1 | 0.399 | 3319 | tags=43%, list=20%, signal=53% |
| LOW_DOSE_B_MALAYI_VS_M_TUBERCULOSIS_MAC_UP | 185 | -0.4207751 | -1.7902877 | 0 | 1 | 0.496 | 3781 | tags=42%, list=22%, signal=54% |
| WT_VS_KLF3_KO_BCELL_DN | 181 | -0.40461457 | -1.7165278 | 0 | 1 | 0.708 | 2883 | tags=30%, list=17%, signal=36% |
| 0H_VS_1H_IN_VITRO_ACT_CD4_TCELL_UP | 151 | -0.40443322 | -1.7114873 | 0 | 1 | 0.717 | 2861 | tags=35%, list=17%, signal=42% |
| UNSTIM_VS_45MIN_LPS_STIM_MACROPHAGE_DN | 171 | -0.36125532 | -1.7010738 | 0 | 1 | 0.75 | 3640 | tags=37%, list=21%, signal=47% |
| CTRL_VS_IGE_STIM_MAST_CELL_DN | 175 | -0.38452482 | -1.6455853 | 0 | 1 | 0.865 | 2775 | tags=31%, list=16%, signal=37% |
| LAIV_VS_TIV_FLU_VACCINE_DAY7_MONOCYTE_DN | 174 | -0.42649364 | -1.7730993 | 0.001904762 | 1 | 0.543 | 3064 | tags=32%, list=18%, signal=38% |
| L_DONOVANI_VS_B_MALAYI_LOW_DOSE_MAC_DN | 180 | -0.42613578 | -1.7056642 | 0.003773585 | 1 | 0.737 | 2452 | tags=34%, list=14%, signal=39% |
| THYMIC_DC_VS_THYMIC_MACROPHAGE_DN | 177 | -0.36213714 | -1.62156 | 0.003937008 | 1 | 0.896 | 2655 | tags=29%, list=16%, signal=34% |
| UNTREATED_VS_A2AR_AGONIST_TREATED_TREG_DN | 180 | -0.39936164 | -1.6302571 | 0.005555556 | 1 | 0.888 | 2883 | tags=37%, list=17%, signal=44% |
| CTRL_VS_M_TUBERCULOSIS_MAC_UP | 178 | -0.42739135 | -1.7569245 | 0.005791506 | 1 | 0.59 | 2997 | tags=39%, list=18%, signal=47% |
| CTRL_VS_16H_IFNG_IN_CD8POS_DC_DN | 173 | -0.4755264 | -1.7656779 | 0.007604563 | 1 | 0.562 | 2306 | tags=36%, list=14%, signal=42% |
| B2_VS_B1_BCELL_DN | 173 | -0.39998096 | -1.6353273 | 0.007874016 | 1 | 0.879 | 2655 | tags=32%, list=16%, signal=38% |
| YOUNG_VS_OLD_DONOR_MEMORY_CD4_TCELL_40H_TSST_ACT_DN | 177 | -0.49443877 | -1.6866304 | 0.007905139 | 1 | 0.788 | 2852 | tags=45%, list=17%, signal=54% |
| EFFECTOR_VS_EXHAUSTED_CD8_TCELL_UP | 176 | -0.48362553 | -1.7393348 | 0.00967118 | 1 | 0.645 | 1594 | tags=33%, list=9%, signal=36% |
| LPS_VS_LPS_AND_IL10_STIM_MACROPHAGE_45MIN_DN | 171 | -0.40406442 | -1.6850973 | 0.009803922 | 1 | 0.793 | 3808 | tags=37%, list=22%, signal=47% |
| CTRL_VS_POLYIC_8H_BMDC_UP | 171 | -0.42854932 | -1.6189181 | 0.00990099 | 1 | 0.902 | 2994 | tags=40%, list=18%, signal=48% |
| CD69_NEG_VS_POS_TREG_CD62L_LOS_KLRG1_NEG_DN | 174 | -0.44141614 | -1.6859571 | 0.011049724 | 1 | 0.791 | 1957 | tags=36%, list=12%, signal=40% |
| NAIVE_VS_MEMORY_CD8_TCELL_DN | 185 | -0.43321946 | -1.6548561 | 0.013333334 | 1 | 0.847 | 2857 | tags=36%, list=17%, signal=42% |
| CTRL_VS_T_GONDII_MAC_UP | 185 | -0.42299473 | -1.6180373 | 0.014953272 | 1 | 0.904 | 2900 | tags=39%, list=17%, signal=47% |
| NAIVE_VS_IGM_MEMORY_BCELL_DN | 175 | -0.42898095 | -1.6663499 | 0.015296367 | 1 | 0.829 | 1571 | tags=29%, list=9%, signal=31% |
| CTRL_VS_L_DONOVANI_MAC_UP | 183 | -0.49010056 | -1.7218539 | 0.015503876 | 1 | 0.691 | 2213 | tags=42%, list=13%, signal=47% |
| KAECH_NAIVE_VS_MEMORY_CD8_TCELL_DN | 185 | -0.4477836 | -1.6560085 | 0.017681729 | 1 | 0.847 | 2857 | tags=36%, list=17%, signal=42% |
| FOLLICULAR_BCELL_VS_MEMORY_BCELL_DAY7_UP | 173 | -0.43440878 | -1.6690058 | 0.01934236 | 1 | 0.822 | 2626 | tags=35%, list=15%, signal=41% |
| SCHERER_PBMC_APSV_WETVAX_AGE_18_32YO_2_TO_4DY_DN | 32 | -0.59861326 | -1.7129974 | 0.027888447 | 1 | 0.715 | 2064 | tags=44%, list=12%, signal=50% |
| OCONNOR_PBMC_MENVEO_ACWYVAX_AGE_30_70YO_7DY_AFTER_SECOND_DOSE_VS_7DY_AFTER_FIRST_DOSE_UP | 120 | -0.49425325 | -1.6942667 | 0.029880479 | 1 | 0.771 | 2243 | tags=42%, list=13%, signal=48% |
| HOFT_CD4_POSITIVE_ALPHA_BETA_MEMORY_T_CELL_BCG_VACCINE_AGE_18_45YO_56D_TOP_100_DEG_AFTER_IN_VITRO_RE_STIMULATION_DN | 42 | -0.5167033 | -1.6490937 | 0.03065134 | 1 | 0.861 | 3644 | tags=50%, list=21%, signal=64% |
| THAKAR_PBMC_INACTIVATED_INFLUENZA_AGE_21_30YO_NONRESPONDER_7DY_DN | 63 | -0.5514914 | -1.6377926 | 0.04255319 | 1 | 0.875 | 2622 | tags=49%, list=15%, signal=58% |
| LI_PBMC_MENOMUNE_A_C_Y_W_135_AGE_18_45YO_CORRELATED_WITH_ANTIBODY_RESPONSE_3DY_POSITIVE | 43 | -0.6333279 | -1.71113 | 0.044989776 | 1 | 0.717 | 2443 | tags=56%, list=14%, signal=65% |
| **JUN** |  |  |  |  |  |  |  |  |
| **Hallmark gene set** |  |  |  |  |  |  |  |  |
| NAME | SIZE | ES | NES | NOM p-val | FDR q-val | FWER p-val | RANK AT MAX | LEADING EDGE |
| HALLMARK_NOTCH_SIGNALING | 32 | 0.47402537 | 1.57973 | 0.032608695 | 0.7130351 | 0.344 | 3678 | tags=50%, list=22%, signal=64% |
| HALLMARK_CHOLESTEROL_HOMEOSTASIS | 72 | 0.39115965 | 1.5486481 | 0.030241935 | 0.44133985 | 0.397 | 2349 | tags=29%, list=14%, signal=34% |
| HALLMARK_DNA_REPAIR | 135 | -0.48690644 | -1.7059531 | 0.0186722 | 0.26214013 | 0.162 | 1593 | tags=39%, list=9%, signal=42% |
| **Immunologic signature gene set** |  |  |  |  |  |  |  |  |
| NAME | SIZE | ES | NES | NOM p-val | FDR q-val | FWER p-val | RANK AT MAX | LEADING EDGE |
| UNTREATED_VS_ACT_CD4_TCELL_48H_DN | 159 | 0.41863275 | 1.7918559 | 0 | 1 | 0.47 | 3192 | tags=34%, list=19%, signal=41% |
| UNTREATED_VS_AD5_T425A_HEXON_INF_MOUSE_LUNG_DC_UP | 176 | 0.36368194 | 1.7438312 | 0 | 1 | 0.613 | 2590 | tags=24%, list=15%, signal=29% |
| PRIMARY_VS_SECONDARY_ACUTE_LCMV_INF_CD8_TCELL_DN | 170 | 0.3552167 | 1.7049977 | 0 | 1 | 0.716 | 2826 | tags=28%, list=17%, signal=34% |
| 0.5H_VS_4H_LPS_BMDC_DN | 178 | 0.36143512 | 1.704019 | 0 | 1 | 0.718 | 2027 | tags=25%, list=12%, signal=28% |
| CTRL_VS_LPS_4H_BMDC_DN | 178 | 0.33793303 | 1.6976942 | 0 | 1 | 0.735 | 2297 | tags=25%, list=14%, signal=29% |
| CTRL_VS_FOXP3_OVEREXPR_TCONV_DN | 167 | 0.3344844 | 1.5882417 | 0 | 1 | 0.925 | 2366 | tags=25%, list=14%, signal=28% |
| PRE_BCELL_VS_SMALL_PRE_BCELL_UP | 141 | 0.34344116 | 1.5845594 | 0 | 1 | 0.928 | 2994 | tags=32%, list=18%, signal=38% |
| 0H_VS_24H_OZONE_TLR4_KO_LUNG_DN | 185 | 0.32112238 | 1.5639207 | 0 | 1 | 0.943 | 2313 | tags=23%, list=14%, signal=27% |
| RETINOIC_ACID_VS_RETINOIC_ACID_AND_PAM2CSK4_STIM_FOLLICULAR_DC_UP | 177 | 0.29626366 | 1.4868215 | 0 | 1 | 0.985 | 3174 | tags=28%, list=19%, signal=34% |
| WT_VS_PPARG_KO_MACROPHAGE_ROSIGLITAZONE_STIM_UP | 174 | 0.33277354 | 1.565421 | 0.002066116 | 1 | 0.943 | 2906 | tags=27%, list=17%, signal=32% |
| TGFB_VS_IL6_TREATED_STAT3_KO_CD4_TCELL_UP | 168 | 0.35650617 | 1.6310122 | 0.002087683 | 1 | 0.861 | 3374 | tags=34%, list=20%, signal=42% |
| C57BL6_VS_NOD_FOXP3_IRES_GFP_TCONV_DN | 145 | 0.32901272 | 1.5222294 | 0.002096436 | 1 | 0.971 | 3321 | tags=32%, list=20%, signal=40% |
| RARA_AGONIST_AM580_VS_AM580_AND_ROSIGLITAZONE_TREATED_DC_UP | 177 | 0.3232976 | 1.4819262 | 0.002169197 | 1 | 0.987 | 1476 | tags=20%, list=9%, signal=22% |
| FRANCO_BLOOD_SANOFI_PASTEUR_SA_INACTIVATED_INFLUENZA_VACCINE_CORRELATED_WITH_ANTIBODY_RESPONSE_AGE_18_40YO_3DY_POSITIVE | 18 | 0.6941916 | 1.8940417 | 0.003875969 | 1 | 0.226 | 1790 | tags=44%, list=11%, signal=50% |
| ARTHRITIC_SYNOVIAL_FLUID_VS_HEALTHY_MACROPHAGE_DN | 130 | 0.35861477 | 1.5707995 | 0.003913894 | 1 | 0.94 | 2254 | tags=25%, list=13%, signal=29% |
| UNTREATED_VS_ACT_CD4_TCELL_6H_DN | 175 | 0.36500597 | 1.6140696 | 0.004 | 1 | 0.894 | 3520 | tags=36%, list=21%, signal=45% |
| CTRL_VS_B_ABORTUS_4H_MAC_CELL_LINE_UP | 178 | 0.3588048 | 1.5899221 | 0.004219409 | 1 | 0.923 | 2022 | tags=25%, list=12%, signal=28% |
| EOS_AND_LEF1_TRANSDUCED_VS_GATA1_AND_SATB1_TRANSDUCED_CD4_TCELL_UP | 179 | 0.32758188 | 1.5332276 | 0.004301075 | 1 | 0.967 | 2986 | tags=32%, list=18%, signal=38% |
| 4D_VS_8D_CULTURE_BM_PROGENITOR_UP | 152 | 0.37697896 | 1.59237 | 0.006147541 | 1 | 0.919 | 3662 | tags=43%, list=22%, signal=54% |
| UNTREATED_VS_AD5_INF_IL1R_KO_MOUSE_LUNG_DC_UP | 181 | 0.31431273 | 1.5131012 | 0.006185567 | 1 | 0.975 | 2965 | tags=24%, list=17%, signal=29% |
| SPLEEN_MONOCYTE_VS_GMCSF_GCSF_BONE_MARROW_UP | 145 | 0.32441652 | 1.4796271 | 0.008016032 | 1 | 0.987 | 3164 | tags=30%, list=19%, signal=37% |
| UNSTIM_VS_2H_R848_DC_UP | 158 | 0.38485026 | 1.5799891 | 0.008130081 | 1 | 0.934 | 2948 | tags=37%, list=17%, signal=45% |
| SPHK1_KO_VS_HTNFA_OVEREXPRESS_ANKLE_UP | 175 | 0.3208858 | 1.5051764 | 0.010330578 | 1 | 0.979 | 2533 | tags=27%, list=15%, signal=31% |
| FOXP3_VS_FOXP3_AND_GATA1_TRANSDUCED_ACTIVATED_CD4_TCELL_DN | 176 | 0.3409925 | 1.5638444 | 0.011904762 | 1 | 0.943 | 3447 | tags=31%, list=20%, signal=39% |
| UNTREATED_VS_IL2_TREATED_CD8_TCELL_DAY6_POST_IMMUNIZATION_DN | 183 | 0.3850516 | 1.6105169 | 0.012578616 | 1 | 0.899 | 4225 | tags=43%, list=25%, signal=56% |
| LPS_VS_LPS_AND_IL10_STIM_IL10_KO_NFKBP50_KO_MACROPHAGE_UP | 178 | 0.3335149 | 1.509973 | 0.012605042 | 1 | 0.976 | 3828 | tags=37%, list=23%, signal=47% |
| LEF1_VS_FOXP3_AND_LEF1_TRANSDUCED_ACTIVATED_CD4_TCELL_DN | 167 | 0.34457418 | 1.4905457 | 0.014 | 1 | 0.985 | 4269 | tags=41%, list=25%, signal=54% |
| IL4_AND_ANTI_IL12_VS_UNTREATED_12H_ACT_CD4_TCELL_UP | 164 | 0.37796906 | 1.5467962 | 0.016 | 1 | 0.959 | 3005 | tags=35%, list=18%, signal=43% |
| UNSTIM_VS_LPS_STIM_VD1_GAMMADELTA_TCELL_DN | 134 | 0.33607775 | 1.508009 | 0.017928287 | 1 | 0.977 | 2064 | tags=23%, list=12%, signal=26% |
| LPS_VS_PAM3CSK4_1H_BMDC_DN | 187 | 0.32302684 | 1.4993871 | 0.018518519 | 1 | 0.983 | 2540 | tags=24%, list=15%, signal=28% |
| IL10_KO_VS_IL10_KO_AND_NFKBP50_KO_LPS_AND_IL10_STIM_MACROPHAGE_UP | 181 | 0.33367687 | 1.4833856 | 0.01863354 | 1 | 0.986 | 3396 | tags=33%, list=20%, signal=41% |
| NAIVE_CD4_TCELL_VS_INDUCED_TREG_UP | 167 | 0.3974716 | 1.5781765 | 0.02016129 | 1 | 0.935 | 3293 | tags=37%, list=19%, signal=46% |
| GERMFREE_VS_SPF_ARTHRITIC_MOUSE_CD4_TCELL_UP | 189 | 0.3480278 | 1.5373081 | 0.020703934 | 1 | 0.965 | 2307 | tags=24%, list=14%, signal=28% |
| UNTREATED_VS_IFNA_TREATED_EPITHELIAL_CELLS_6H_DN | 166 | 0.43631876 | 1.6268115 | 0.023483366 | 1 | 0.868 | 3699 | tags=42%, list=22%, signal=53% |
| LPS_VS_POLYIC_4H_BMDC_UP | 190 | 0.34943992 | 1.4994808 | 0.026970955 | 1 | 0.983 | 3430 | tags=34%, list=20%, signal=42% |
| UNTREATED_VS_IL2_TREATED_CD8_TCELL_DAY3_POST_IMMUNIZATION_UP | 178 | 0.34948856 | 1.4903123 | 0.031055901 | 1 | 0.985 | 3297 | tags=28%, list=19%, signal=34% |
| UNSTIM_VS_2H_LPS_DC_UP | 149 | 0.37600723 | 1.507312 | 0.0332681 | 1 | 0.977 | 4010 | tags=41%, list=24%, signal=53% |
| 2H_VS_72H_UNTREATED_IN_VITRO_CD4_TCELL_UP | 166 | 0.40541643 | 1.5346599 | 0.034548946 | 1 | 0.966 | 2987 | tags=37%, list=18%, signal=45% |
| FRANCO_BLOOD_SANOFI_PASTEUR_SA_INACTIVATED_INFLUENZA_VACCINE_CORRELATED_WITH_ANTIBODY_RESPONSE_AGE_18_40YO_1DY_NEGATIVE | 18 | 0.656322 | 1.6474074 | 0.037924152 | 1 | 0.832 | 2231 | tags=61%, list=13%, signal=70% |
| ERWIN_COHEN_PBMC_TC_83_AGE_18_45YO_NON_RESPONDERS_PREVIOUSLY_IMMUNIZED_24HR_DEG_CANONICAL_PATHWAY_MEMBERS_UP | 18 | 0.5942673 | 1.5796827 | 0.03883495 | 1 | 0.935 | 1139 | tags=33%, list=7%, signal=36% |
| DAY6_EFF_VS_DAY150_MEM_LY6C_INT_CXCR5POS_CD4_TCELL_UP | 173 | 0.360688 | 1.4990857 | 0.047227927 | 1 | 0.983 | 2953 | tags=30%, list=17%, signal=36% |
| UNTREATED_VS_IFNG_TREATED_EPITHELIAL_CELLS_24H_DN | 165 | 0.38176656 | 1.5121953 | 0.04771372 | 1 | 0.975 | 4493 | tags=43%, list=26%, signal=58% |
| HEALTHY_VS_MCMV_INFECTION_CD11B_DC_IFNAR_KO_UP | 164 | 0.37589207 | 1.4892876 | 0.048879836 | 1 | 0.985 | 3355 | tags=35%, list=20%, signal=44% |
| HOFT_CD4_POSITIVE_ALPHA_BETA_MEMORY_T_CELL_BCG_VACCINE_AGE_18_45YO_56D_TOP_100_DEG_AFTER_IN_VITRO_RE_STIMULATION_UP | 16 | 0.64947873 | 1.6136742 | 0.0498008 | 1 | 0.895 | 1727 | tags=44%, list=10%, signal=49% |
| IL12_VS_IL4_TREATED_ACT_CD4_TCELL_6H_UP | 175 | -0.37012583 | -1.5949622 | 0.001941748 | 1 | 0.938 | 2314 | tags=27%, list=14%, signal=31% |
| UNSTIM_VS_45MIN_LPS_STIM_MACROPHAGE_DN | 171 | -0.31923825 | -1.4721951 | 0.003853565 | 1 | 0.993 | 2263 | tags=24%, list=13%, signal=27% |
| DOUBLE_NEGATIVE_VS_DOUBLE_POSITIVE_THYMOCYTE_UP | 158 | -0.30789676 | -1.4868401 | 0.005714286 | 1 | 0.991 | 3474 | tags=28%, list=20%, signal=35% |
| T_GONDII_VS_B_MALAYI_LOW_DOSE_MAC_UP | 175 | -0.3694436 | -1.6187127 | 0.007532957 | 1 | 0.906 | 3123 | tags=35%, list=18%, signal=42% |
| THYMIC_DC_VS_THYMIC_MACROPHAGE_DN | 177 | -0.3631511 | -1.5710078 | 0.007707129 | 1 | 0.962 | 1878 | tags=24%, list=11%, signal=27% |
| B2_VS_B1_BCELL_DN | 173 | -0.4135902 | -1.6338372 | 0.013108614 | 1 | 0.888 | 2266 | tags=31%, list=13%, signal=35% |
| CTRL_VS_PAM3CSK4_0.5H_BMDC_DN | 178 | -0.31820878 | -1.4691567 | 0.014814815 | 1 | 0.994 | 2545 | tags=25%, list=15%, signal=29% |
| LAIV_VS_TIV_FLU_VACCINE_DAY7_MONOCYTE_DN | 174 | -0.3846561 | -1.5345275 | 0.01663586 | 1 | 0.981 | 2544 | tags=28%, list=15%, signal=32% |
| CD4_TCELL_BALBC_VS_TH17_ENRI_CD4_TCELL_SKG_PMA_IONO_STIM_FR4NEG_UP | 177 | -0.34254137 | -1.5115258 | 0.018691588 | 1 | 0.989 | 2260 | tags=22%, list=13%, signal=25% |
| BCL6_LOW_TFH_VS_NAIVE_CD4_TCELL_UP | 183 | -0.35711804 | -1.4734803 | 0.026266417 | 1 | 0.993 | 3043 | tags=32%, list=18%, signal=39% |
| 4D_VS_8D_CULTURE_MIR223_KO_BM_PROGENITOR_DN | 148 | -0.36087042 | -1.466496 | 0.028462999 | 1 | 0.994 | 2651 | tags=32%, list=16%, signal=37% |
| DC_VS_MAC_DN | 181 | -0.3631974 | -1.4643117 | 0.03538175 | 1 | 0.994 | 3931 | tags=39%, list=23%, signal=50% |
| SOCS3_KO_VS_IFNG_KO_LIVER_DN | 171 | -0.32017684 | -1.4668901 | 0.040076334 | 1 | 0.994 | 3165 | tags=29%, list=19%, signal=35% |
| CD161_HIGH_VS_INT_CD8_TCELL_DN | 170 | -0.40704465 | -1.5882517 | 0.041015625 | 1 | 0.947 | 2387 | tags=32%, list=14%, signal=37% |
| CD4_TCELL_VS_NKT_CELL_DN | 171 | -0.37412906 | -1.5183064 | 0.046153847 | 1 | 0.989 | 4142 | tags=44%, list=24%, signal=58% |
| CTRL_VS_L_DONOVANI_MAC_UP | 183 | -0.44248605 | -1.5155259 | 0.046904314 | 1 | 0.989 | 2753 | tags=38%, list=16%, signal=45% |
| **PTMA** |  |  |  |  |  |  |  |  |
| **Hallmark gene set** |  |  |  |  |  |  |  |  |
| NAME | SIZE | ES | NES | NOM p-val | FDR q-val | FWER p-val | RANK AT MAX | LEADING EDGE |
| HALLMARK_CHOLESTEROL_HOMEOSTASIS | 72 | 0.41908202 | 1.6558524 | 0.012320329 | 0.1967225 | 0.199 | 2546 | tags=35%, list=15%, signal=41% |
| **Immunologic signature gene set** |  |  |  |  |  |  |  |  |
| NAME | SIZE | ES | NES | NOM p-val | FDR q-val | FWER p-val | RANK AT MAX | LEADING EDGE |
| ANDERSON_BLOOD_CN54GP140_ADJUVANTED_WITH_GLA_AF_AGE_18_45YO_1DY_UP | 73 | 0.5420078 | 1.9292822 | 0 | 0.59251153 | 0.156 | 2337 | tags=41%, list=14%, signal=47% |
| EFFECTOR_VS_EXHAUSTED_CD8_TCELL_DN | 176 | 0.3106436 | 1.5404347 | 0 | 1 | 0.972 | 3892 | tags=31%, list=23%, signal=40% |
| PHD3_KO_VS_WT_NEUTROPHIL_HYPOXIA_DN | 181 | 0.3047981 | 1.497847 | 0.002008032 | 1 | 0.993 | 2726 | tags=23%, list=16%, signal=27% |
| STIM_VS_STIM_AND_TRICHOSTATINA_72H_CD8_T_CELL_DN | 183 | 0.2995484 | 1.4421964 | 0.004 | 1 | 0.999 | 3430 | tags=30%, list=20%, signal=37% |
| CTRL_VS_LPS_4H_BMDC_DN | 178 | 0.28250498 | 1.413547 | 0.004016064 | 1 | 1 | 3694 | tags=30%, list=22%, signal=38% |
| UNSTIM_VS_LPS_AND_IL10_STIM_IL10_KO_NFKBP50_KO_MACROPHAGE_UP | 157 | 0.34306955 | 1.55203 | 0.004056795 | 1 | 0.964 | 4762 | tags=45%, list=28%, signal=61% |
| FRANCO_BLOOD_SANOFI_PASTEUR_SA_INACTIVATED_INFLUENZA_VACCINE_CORRELATED_WITH_ANTIBODY_RESPONSE_AGE_18_40YO_1DY_NEGATIVE | 18 | 0.7367858 | 1.8213173 | 0.005649718 | 1 | 0.406 | 1545 | tags=61%, list=9%, signal=67% |
| COMMON_LYMPHOID_PROGENITOR_VS_PRO_BCELL_UP | 177 | 0.31205672 | 1.504796 | 0.006437768 | 1 | 0.991 | 2343 | tags=22%, list=14%, signal=25% |
| HOFT_CD4_POSITIVE_ALPHA_BETA_MEMORY_T_CELL_BCG_VACCINE_AGE_18_45YO_56D_TOP_100_DEG_AFTER_IN_VITRO_RE_STIMULATION_UP | 16 | 0.71245515 | 1.721374 | 0.00750469 | 1 | 0.662 | 2484 | tags=69%, list=15%, signal=80% |
| PRIMARY_VS_SECONDARY_ACUTE_LCMV_INF_CD8_TCELL_DN | 170 | 0.30324656 | 1.4754933 | 0.008510638 | 1 | 0.997 | 2107 | tags=22%, list=12%, signal=25% |
| CTRL_VS_FOXP3_OVEREXPR_TCONV_DN | 167 | 0.3066728 | 1.4896246 | 0.010593221 | 1 | 0.996 | 3386 | tags=31%, list=20%, signal=39% |
| IL1B_IL6_VS_IL1B_IL6_IL23A_TREATED_CD4_TCELL_DN | 176 | 0.3025095 | 1.4212265 | 0.012552301 | 1 | 1 | 2811 | tags=27%, list=17%, signal=32% |
| UNSTIM_VS_ANTI_CD3_STIM_DP_THYMOCYTES_UP | 166 | 0.33803326 | 1.5506085 | 0.012658228 | 1 | 0.965 | 3265 | tags=34%, list=19%, signal=42% |
| CTRL_VS_PAM3CSK4_4H_BMDC_DN | 176 | 0.2647998 | 1.3508755 | 0.012738854 | 1 | 1 | 2735 | tags=24%, list=16%, signal=28% |
| HEALTHY_VS_TUMOR_BEARING_MOUSE_SPLEEN_MONOCYTE_24H_INCUBATION_UP | 167 | 0.26759252 | 1.3343766 | 0.012765957 | 1 | 1 | 4027 | tags=30%, list=24%, signal=39% |
| ANDERSON_BLOOD_CN54GP140_ADJUVANTED_WITH_GLA_AF_AGE_18_45YO_7DY_UP | 30 | 0.59999174 | 1.6644958 | 0.01369863 | 1 | 0.798 | 2257 | tags=50%, list=13%, signal=58% |
| SUBCAPSULAR_CORTICAL_REGION_VS_WHOLE_CORTEX_THYMUS_UP | 186 | 0.30442247 | 1.4263096 | 0.014344262 | 1 | 1 | 1634 | tags=20%, list=10%, signal=22% |
| MEMORY_BCELL_DAY7_VS_MEMORY_BCELL_DAY40_DN | 177 | 0.28422317 | 1.3874457 | 0.017391304 | 1 | 1 | 2717 | tags=24%, list=16%, signal=29% |
| CTRL_VS_B_ABORTUS_4H_MAC_CELL_LINE_UP | 178 | 0.33669528 | 1.5131884 | 0.018255578 | 1 | 0.989 | 1568 | tags=21%, list=9%, signal=23% |
| CD103_POS_VS_CD103_KLRG1_DP_TREG_DN | 184 | 0.28203803 | 1.3558898 | 0.01863354 | 1 | 1 | 3839 | tags=31%, list=23%, signal=40% |
| ANTI_CD3CD28_STIM_VS_UNSTIM_MEMORY_CD8_TCELL_UP | 180 | 0.31926158 | 1.4633139 | 0.021231422 | 1 | 0.998 | 2731 | tags=27%, list=16%, signal=31% |
| TREG_VS_TCONV_UP | 174 | 0.30890268 | 1.426532 | 0.021868788 | 1 | 1 | 3127 | tags=27%, list=18%, signal=33% |
| THPOK_KO_VS_WT_VA14I_NKTCELL_UP | 175 | 0.2802314 | 1.3684547 | 0.023076924 | 1 | 1 | 4806 | tags=37%, list=28%, signal=51% |
| CTRL_VS_HCMV_INF_MONOCYTES_DN | 150 | 0.28608146 | 1.3558934 | 0.023762377 | 1 | 1 | 1857 | tags=17%, list=11%, signal=19% |
| CTRL_VS_ANTI_IGM_STIM_ZFX_KO_BCELL_12H_UP | 130 | 0.351653 | 1.4904099 | 0.024291499 | 1 | 0.996 | 2128 | tags=22%, list=13%, signal=25% |
| 33D1_POS_DC_VS_BCELL_UP | 144 | 0.30312023 | 1.3836726 | 0.028806584 | 1 | 1 | 3504 | tags=27%, list=21%, signal=34% |
| PRE_VS_DAY7_POST_TIV_FLU_VACCINE_MDC_DN | 167 | 0.271272 | 1.3429692 | 0.028806584 | 1 | 1 | 1812 | tags=20%, list=11%, signal=23% |
| UNTREATED_VS_AD5_T425A_HEXON_INF_IL1R_KO_MOUSE_LUNG_DC_DN | 176 | 0.29177842 | 1.3736352 | 0.028901733 | 1 | 1 | 4926 | tags=38%, list=29%, signal=53% |
| HEALTHY_VS_TYPE_1_DIABETES_PBMC_1MONTH_POST_DX_DN | 171 | 0.2966305 | 1.3803685 | 0.029239766 | 1 | 1 | 3282 | tags=27%, list=19%, signal=34% |
| IL2_ACT_IL2_STARVED_VS_IL21_ACT_IL2_STARVED_CD4_TCELL_DN | 131 | 0.28981486 | 1.3897928 | 0.032520324 | 1 | 1 | 1891 | tags=19%, list=11%, signal=21% |
| CPG_VS_GARDIQUIMOD_16H_BMDC_DN | 183 | 0.28730252 | 1.3570948 | 0.034136545 | 1 | 1 | 2138 | tags=21%, list=13%, signal=24% |
| WT_VS_PPARG_KO_MACROPHAGE_ROSIGLITAZONE_STIM_UP | 174 | 0.28994712 | 1.3668191 | 0.039045554 | 1 | 1 | 3293 | tags=27%, list=19%, signal=33% |
| CTRL_VS_IL4_STIM_STAT6_KO_MACROPHAGE_UP | 152 | 0.28026754 | 1.3360207 | 0.039832287 | 1 | 1 | 3172 | tags=26%, list=19%, signal=31% |
| ARTHRITIC_SYNOVIAL_FLUID_VS_HEALTHY_MACROPHAGE_DN | 130 | 0.31524596 | 1.3917363 | 0.040733196 | 1 | 1 | 2745 | tags=26%, list=16%, signal=31% |
| NOD2_TRANSD_VS_CTRL_TRANSD_HEK293_MDP_STIM_2H_UP | 170 | 0.29873872 | 1.3517059 | 0.041420117 | 1 | 1 | 3329 | tags=24%, list=20%, signal=30% |
| ANDERSON_BLOOD_CN54GP140_ADJUVANTED_WITH_GLA_AF_AGE_18_45YO_3DY_UP | 24 | 0.5142682 | 1.4910864 | 0.04901961 | 1 | 0.996 | 2257 | tags=38%, list=13%, signal=43% |
| LOW_DOSE_B_MALAYI_VS_M_TUBERCULOSIS_MAC_UP | 185 | -0.44141906 | -1.8231916 | 0 | 1 | 0.416 | 2542 | tags=34%, list=15%, signal=39% |
| MARGINAL_ZONE_BCELL_VS_FOLLICULAR_BCELL_IRF8_KO_UP | 178 | -0.31503144 | -1.5544466 | 0.001879699 | 1 | 0.964 | 3237 | tags=28%, list=19%, signal=34% |
| CTRL_VS_M_TUBERCULOSIS_MAC_UP | 178 | -0.44185337 | -1.7845652 | 0.001886793 | 1 | 0.53 | 1911 | tags=30%, list=11%, signal=34% |
| CTRL_VS_ANTI_VALPHA2_DP_THYMOCYTE_UP | 179 | -0.33832502 | -1.5620949 | 0.00203666 | 1 | 0.959 | 2909 | tags=28%, list=17%, signal=33% |
| UNINF_VS_LISTERIA_INFECTED_MACROPHAGE_DN | 175 | -0.349375 | -1.6065999 | 0.005586592 | 1 | 0.921 | 3602 | tags=34%, list=21%, signal=42% |
| CTRL_VS_A3R_ACTIVATION_MAST_CELL_UP | 175 | -0.34768078 | -1.5850693 | 0.005780347 | 1 | 0.94 | 4822 | tags=45%, list=28%, signal=62% |
| WT_VS_KLF3_KO_BCELL_DN | 181 | -0.38164055 | -1.6301107 | 0.00750469 | 1 | 0.89 | 2561 | tags=27%, list=15%, signal=31% |
| T_GONDII_VS_B_MALAYI_LOW_DOSE_MAC_UP | 175 | -0.35964966 | -1.5867577 | 0.007782101 | 1 | 0.938 | 2856 | tags=31%, list=17%, signal=37% |
| MAC_VS_BCELL_DN | 167 | -0.4140624 | -1.6655279 | 0.009380863 | 1 | 0.847 | 3048 | tags=37%, list=18%, signal=44% |
| LIN_NEG_VS_NKTCELL_DN | 175 | -0.38214725 | -1.5639302 | 0.00967118 | 1 | 0.959 | 2119 | tags=29%, list=12%, signal=33% |
| 3H_VS_24H_POLYIC_STIM_DC_UP | 143 | -0.4045809 | -1.650134 | 0.011009174 | 1 | 0.869 | 2777 | tags=32%, list=16%, signal=38% |
| LPS_VS_LPS_AND_IL10_STIM_MACROPHAGE_45MIN_DN | 171 | -0.392556 | -1.6264814 | 0.011152417 | 1 | 0.896 | 2769 | tags=30%, list=16%, signal=35% |
| DP_VS_CD8_SP_THYMOCYTE_DN | 180 | -0.34575948 | -1.5201757 | 0.011406845 | 1 | 0.982 | 2143 | tags=30%, list=13%, signal=34% |
| THYMIC_DC_VS_THYMIC_MACROPHAGE_DN | 177 | -0.35091037 | -1.5519539 | 0.012195122 | 1 | 0.966 | 2845 | tags=30%, list=17%, signal=36% |
| WT_VS_KLF2_KO_LPS_STIM_MACROPHAGE_UP | 181 | -0.344255 | -1.529219 | 0.013307985 | 1 | 0.977 | 3389 | tags=33%, list=20%, signal=41% |
| HIGH_IL7_VS_HIGH_IL7_AND_IRF4_IN_IRF4_8_NULL_PRE_BCELL_DN | 177 | -0.3740583 | -1.6261536 | 0.015686275 | 1 | 0.897 | 3095 | tags=34%, list=18%, signal=41% |
| DC_VS_MAC_DN | 181 | -0.40642115 | -1.6600615 | 0.016363636 | 1 | 0.853 | 3113 | tags=35%, list=18%, signal=42% |
| ANDERSON_BLOOD_CN54GP140_ADJUVANTED_WITH_GLA_AF_AGE_18_45YO_1DY_DN | 76 | -0.64810944 | -1.7319883 | 0.016393442 | 1 | 0.686 | 2724 | tags=67%, list=16%, signal=80% |
| EFFECTOR_VS_EXHAUSTED_CD8_TCELL_UP | 176 | -0.4843623 | -1.7218904 | 0.016513761 | 1 | 0.71 | 2620 | tags=39%, list=15%, signal=45% |
| 0H_VS_1H_IN_VITRO_ACT_CD4_TCELL_UP | 151 | -0.38038978 | -1.5818563 | 0.016917294 | 1 | 0.942 | 3292 | tags=36%, list=19%, signal=44% |
| PPARG_LIGAND_ROSIGLITAZONE_VS_RARA_AGONIST_AM580_TREATED_DC_DN | 180 | -0.3575307 | -1.54832 | 0.018348623 | 1 | 0.967 | 1717 | tags=23%, list=10%, signal=25% |
| CTRL_VS_L_DONOVANI_MAC_UP | 183 | -0.48913231 | -1.7231827 | 0.020295203 | 1 | 0.707 | 2790 | tags=44%, list=16%, signal=52% |
| THAKAR_PBMC_INACTIVATED_INFLUENZA_AGE_21_30YO_NONRESPONDER_7DY_DN | 63 | -0.57386446 | -1.6892009 | 0.02037037 | 1 | 0.796 | 1776 | tags=43%, list=10%, signal=48% |
| DP_VS_CD4_THYMOCYTE_UP | 175 | -0.38806155 | -1.5672206 | 0.020952381 | 1 | 0.959 | 3855 | tags=41%, list=23%, signal=52% |
| DAY8_EFFECTOR_VS_DAY30_MEMORY_CD8_TCELL_LCMV_ARMSTRONG_DN | 177 | -0.3913141 | -1.5280087 | 0.021072797 | 1 | 0.978 | 3105 | tags=36%, list=18%, signal=43% |
| 0.5H_VS_8H_CPG_BMDC_UP | 177 | -0.43578222 | -1.6001276 | 0.031307552 | 1 | 0.933 | 2911 | tags=41%, list=17%, signal=49% |
| DC_VS_MAC_B_MALAYI_LOW_DOSE_UP | 178 | -0.38719597 | -1.547619 | 0.031835206 | 1 | 0.967 | 2631 | tags=35%, list=15%, signal=41% |
| OCONNOR_PBMC_MENVEO_ACWYVAX_AGE_30_70YO_7DY_AFTER_SECOND_DOSE_VS_7DY_AFTER_FIRST_DOSE_UP | 120 | -0.48293072 | -1.6458662 | 0.032323234 | 1 | 0.874 | 1941 | tags=38%, list=11%, signal=42% |
| GMCSF_AND_CURDLAN_LOWDOSE_VS_GMCSF_AND_CURDLAN_HIGHDOSE_STIM_DC_UP | 175 | -0.40674543 | -1.5584598 | 0.033395175 | 1 | 0.961 | 3112 | tags=37%, list=18%, signal=44% |
| CD4_TCELL_VS_B2_BCELL_DN | 175 | -0.39497226 | -1.5795221 | 0.033898305 | 1 | 0.945 | 2844 | tags=37%, list=17%, signal=43% |
| FOXO1_KO_VS_WT_NTREG_UP | 172 | -0.41530868 | -1.536569 | 0.03409091 | 1 | 0.972 | 3207 | tags=38%, list=19%, signal=46% |
| UNINF_VS_LEISHMANIA_INF_DC_24H_DN | 184 | -0.34386635 | -1.5381588 | 0.034883723 | 1 | 0.972 | 3401 | tags=35%, list=20%, signal=44% |
| UNINF_VS_LEISHMANIA_INF_DC_2H_UP | 176 | -0.3604778 | -1.5390525 | 0.036072146 | 1 | 0.972 | 3401 | tags=36%, list=20%, signal=44% |
| CTRL_VS_16H_IFNG_IN_CD8POS_DC_DN | 173 | -0.43967965 | -1.6049173 | 0.039848197 | 1 | 0.924 | 3145 | tags=42%, list=19%, signal=51% |
| MAC_VS_TH2_DN | 178 | -0.39702365 | -1.5743867 | 0.04191617 | 1 | 0.949 | 3149 | tags=37%, list=19%, signal=45% |
| PRIMARY_VS_TERTIARY_MEMORY_CD8_TCELL_UP | 187 | -0.42995983 | -1.5639356 | 0.0433145 | 1 | 0.959 | 2935 | tags=39%, list=17%, signal=46% |
| WT_VS_HEB_KO_DP_THYMOCYTE_DN | 177 | -0.42918837 | -1.5620145 | 0.043643262 | 1 | 0.959 | 2305 | tags=32%, list=14%, signal=37% |
| NAIVE_VS_IGM_MEMORY_BCELL_DN | 175 | -0.4132542 | -1.5715011 | 0.045454547 | 1 | 0.952 | 1811 | tags=29%, list=11%, signal=32% |
| CTRL_VS_T_GONDII_MAC_UP | 185 | -0.41446775 | -1.555842 | 0.04612546 | 1 | 0.963 | 3018 | tags=36%, list=18%, signal=44% |
| 0.5H_VS_8H_POLYIC_BMDC_UP | 180 | -0.39869338 | -1.5192893 | 0.046242774 | 1 | 0.982 | 3707 | tags=39%, list=22%, signal=50% |
| B2_VS_B1_BCELL_DN | 173 | -0.37243256 | -1.5170045 | 0.04733728 | 1 | 0.984 | 2443 | tags=30%, list=14%, signal=35% |
| **SH3YL1** |  |  |  |  |  |  |  |  |
| **Hallmark gene set** |  |  |  |  |  |  |  |  |
| NAME | SIZE | ES | NES | NOM p-val | FDR q-val | FWER p-val | RANK AT MAX | LEADING EDGE |
| HALLMARK_XENOBIOTIC_METABOLISM | 197 | -0.35463163 | -1.521915 | 0.028169014 | 1 | 0.458 | 4982 | tags=45%, list=29%, signal=62% |
| HALLMARK_COAGULATION | 136 | -0.3895866 | -1.508694 | 0.031055901 | 0.921059 | 0.489 | 4457 | tags=40%, list=26%, signal=54% |
| **Immunologic signature gene set** |  |  |  |  |  |  |  |  |
| NAME | SIZE | ES | NES | NOM p-val | FDR q-val | FWER p-val | RANK AT MAX | LEADING EDGE |
| CD45RA_NEG_CD4_TCELL_VS_RESTING_TREG_DN | 169 | 0.35898855 | 1.6468744 | 0 | 1 | 0.864 | 3267 | tags=31%, list=19%, signal=38% |
| BALBC_VS_C57BL6_MONOCYTE_SPLEEN_DN | 146 | 0.34839073 | 1.5343144 | 0 | 1 | 0.979 | 2315 | tags=26%, list=14%, signal=30% |
| UNTREATED_VS_AD5_T425A_HEXON_INF_MOUSE_LUNG_DC_UP | 176 | 0.32335404 | 1.5174807 | 0 | 1 | 0.988 | 2885 | tags=26%, list=17%, signal=30% |
| ARTHRITIC_SYNOVIAL_FLUID_VS_HEALTHY_MACROPHAGE_DN | 130 | 0.38471845 | 1.6788241 | 0.001912046 | 1 | 0.79 | 3035 | tags=31%, list=18%, signal=37% |
| UNTREATED_VS_ACT_CD4_TCELL_6H_DN | 175 | 0.35929143 | 1.5544735 | 0.002008032 | 1 | 0.967 | 2777 | tags=29%, list=16%, signal=34% |
| ANDERSON_BLOOD_CN54GP140_ADJUVANTED_WITH_GLA_AF_AGE_18_45YO_1DY_UP | 73 | 0.49269962 | 1.7939713 | 0.002016129 | 0.8503544 | 0.497 | 3231 | tags=48%, list=19%, signal=59% |
| FRANCO_BLOOD_SANOFI_PASTEUR_SA_INACTIVATED_INFLUENZA_VACCINE_CORRELATED_WITH_ANTIBODY_RESPONSE_AGE_18_40YO_1DY_NEGATIVE | 18 | 0.754597 | 1.8786024 | 0.003597122 | 1 | 0.296 | 2301 | tags=67%, list=14%, signal=77% |
| LEF1_VS_FOXP3_AND_LEF1_TRANSDUCED_ACTIVATED_CD4_TCELL_DN | 167 | 0.37225822 | 1.6009921 | 0.005836576 | 1 | 0.927 | 3104 | tags=34%, list=18%, signal=41% |
| PD1HIGH_VS_PD1LOW_CD8_TCELL_UP | 154 | 0.3677014 | 1.5891703 | 0.005859375 | 1 | 0.941 | 2440 | tags=27%, list=14%, signal=32% |
| ANDERSON_BLOOD_CN54GP140_ADJUVANTED_WITH_GLA_AF_AGE_18_45YO_7DY_UP | 30 | 0.64593834 | 1.8619459 | 0.006024096 | 0.9441421 | 0.339 | 1879 | tags=53%, list=11%, signal=60% |
| CTRL_VS_ANTI_IGM_STIM_BCELL_3H_UP | 156 | 0.36647382 | 1.6517289 | 0.006198347 | 1 | 0.859 | 2414 | tags=28%, list=14%, signal=32% |
| CENT_MEMORY_CD4_TCELL_VS_NKCELL_UP | 175 | 0.41793758 | 1.7082974 | 0.006224067 | 1 | 0.732 | 3302 | tags=41%, list=19%, signal=51% |
| TFH_VS_NON_TFH_CD4_TCELL_UP | 172 | 0.36585695 | 1.5998644 | 0.007692308 | 1 | 0.929 | 2936 | tags=31%, list=17%, signal=38% |
| LPS_VS_PMA_STIM_VD2_GAMMADELTA_TCELL_UP | 115 | 0.34254643 | 1.5186155 | 0.007692308 | 1 | 0.987 | 3980 | tags=41%, list=23%, signal=53% |
| EX_VIVO_VS_DEC205_CONVERSION_DN | 173 | 0.3753972 | 1.6068058 | 0.007936508 | 1 | 0.923 | 2808 | tags=32%, list=17%, signal=38% |
| MED_VS_SCS_MAC_LN_DN | 173 | 0.39521036 | 1.6515901 | 0.011627907 | 1 | 0.859 | 2516 | tags=31%, list=15%, signal=36% |
| LYMPHOID_MULTIPOTENT_VS_MEGAKARYOCYTE_ERYTHROID_PROGENITOR_IKAROS_KO_UP | 152 | 0.3848184 | 1.6133621 | 0.01417004 | 1 | 0.918 | 3473 | tags=36%, list=20%, signal=45% |
| NAIVE_CD4_TCELL_VS_INDUCED_TREG_UP | 167 | 0.4009674 | 1.5772883 | 0.015238095 | 1 | 0.951 | 2868 | tags=35%, list=17%, signal=41% |
| THAKAR_PBMC_INACTIVATED_INFLUENZA_AGE_70PLS_NONRESPONDER_2DY_UP | 93 | 0.68110394 | 1.807705 | 0.015296367 | 0.90865624 | 0.467 | 2806 | tags=60%, list=17%, signal=72% |
| KANNAN_BLOOD_2012_2013_TIV_AGE_65PLS_REVACCINATED_IN_6_9_MO_VS_REVACCINATED_IN_12_13_MO_UP | 184 | 0.52628565 | 1.8292395 | 0.015414258 | 0.9466672 | 0.414 | 3393 | tags=48%, list=20%, signal=59% |
| ADENOSINE_A3R_ACT_VS_TCELL_MEMBRANES_ACT_IN_MAST_CELL_DN | 146 | 0.36690658 | 1.5427753 | 0.015936255 | 1 | 0.975 | 3127 | tags=35%, list=18%, signal=42% |
| CTRL_VS_ANTI_IGM_STIM_ZFX_KO_BCELL_12H_UP | 130 | 0.3645701 | 1.5577878 | 0.016917294 | 1 | 0.965 | 1550 | tags=21%, list=9%, signal=23% |
| HEALTHY_VS_MCMV_INFECTION_CD11B_DC_IFNAR_KO_UP | 164 | 0.4035557 | 1.6068724 | 0.017681729 | 1 | 0.923 | 3357 | tags=38%, list=20%, signal=47% |
| DAY3_VS_DAY6_POST_IMMUNIZATION_CD8_TCELL_UP | 171 | 0.39251083 | 1.5767256 | 0.017892644 | 1 | 0.951 | 2754 | tags=33%, list=16%, signal=39% |
| NAIVE_VS_ACT_CD8_TCELL_DN | 159 | 0.43927112 | 1.6622348 | 0.017964073 | 1 | 0.83 | 3473 | tags=40%, list=20%, signal=49% |
| HOFT_CD4_POSITIVE_ALPHA_BETA_MEMORY_T_CELL_BCG_VACCINE_AGE_18_45YO_56D_TOP_100_DEG_AFTER_IN_VITRO_RE_STIMULATION_UP | 16 | 0.7039942 | 1.7414038 | 0.01831502 | 1 | 0.638 | 1957 | tags=63%, list=12%, signal=71% |
| CTRL_VS_ANTI_IGM_STIM_ZFX_KO_BCELL_12H_DN | 145 | 0.36027846 | 1.547971 | 0.018329939 | 1 | 0.97 | 3443 | tags=34%, list=20%, signal=43% |
| UNTREATED_VS_TGFB_IL6_TREATED_CD4_TCELL_DN | 157 | 0.45158628 | 1.7140944 | 0.019607844 | 1 | 0.717 | 3199 | tags=41%, list=19%, signal=51% |
| UNTREATED_VS_IFNA_AND_IFNG_TREATED_EPITHELIAL_CELLS_6H_UP | 165 | 0.38070497 | 1.5600843 | 0.019880716 | 1 | 0.963 | 3129 | tags=32%, list=18%, signal=39% |
| HSC_VS_MEGAKARYOCYTE_ERYTHROID_PROGENITOR_UP | 146 | 0.40553957 | 1.6232717 | 0.02357564 | 1 | 0.902 | 3473 | tags=36%, list=20%, signal=45% |
| LIGHT_ZONE_VS_NAIVE_BCELL_DN | 164 | 0.37333488 | 1.5158583 | 0.024528302 | 1 | 0.989 | 2833 | tags=31%, list=17%, signal=37% |
| FOURATI_BLOOD_TWINRIX_AGE_25_83YO_RESPONDERS_VS_POOR_RESPONDERS_0DY_UP | 492 | 0.4149689 | 1.6580945 | 0.025793651 | 1 | 0.842 | 3646 | tags=39%, list=21%, signal=48% |
| EFF_MEMORY_VS_CXCR5_POS_CD4_TCELL_DN | 150 | 0.41432682 | 1.5894787 | 0.026476579 | 1 | 0.941 | 3362 | tags=37%, list=20%, signal=45% |
| DAY3_VS_DAY7_LAIV_FLU_VACCINE_PBMC_DN | 160 | 0.35160056 | 1.5127835 | 0.026694044 | 1 | 0.99 | 3900 | tags=33%, list=23%, signal=42% |
| WT_VS_DICER_KO_TREG_DN | 139 | 0.39795473 | 1.5362004 | 0.027131783 | 1 | 0.979 | 3497 | tags=42%, list=21%, signal=52% |
| NAIVE_VS_CENT_MEMORY_CD4_TCELL_UP | 144 | 0.37902158 | 1.5309181 | 0.028056113 | 1 | 0.98 | 3129 | tags=34%, list=18%, signal=41% |
| WT_VS_PPARG_KO_MACROPHAGE_IL4_AND_ROSIGLITAZONE_STIM_DN | 169 | 0.43975165 | 1.5727615 | 0.03006012 | 1 | 0.954 | 2327 | tags=33%, list=14%, signal=38% |
| CD4_TCELL_VS_NEUTROPHIL_UP | 172 | 0.39169234 | 1.5176208 | 0.031311154 | 1 | 0.988 | 2637 | tags=35%, list=16%, signal=41% |
| BALBC_VS_C57BL6_MONOCYTE_TUMOR_DN | 153 | 0.3761705 | 1.529032 | 0.031835206 | 1 | 0.982 | 3004 | tags=34%, list=18%, signal=41% |
| RESTING_VS_BYSTANDER_ACTIVATED_CD4_TCELL_UP | 139 | 0.4247729 | 1.5847613 | 0.032520324 | 1 | 0.946 | 2658 | tags=34%, list=16%, signal=40% |
| WT_VS_IFNAR_KO_CD11B_DC_UP | 164 | 0.41547832 | 1.5956597 | 0.03269231 | 1 | 0.933 | 3592 | tags=45%, list=21%, signal=56% |
| UNSTIM_VS_MCSF_TREATED_MONOCYTE_DAY3_UP | 175 | 0.387088 | 1.5225635 | 0.033398822 | 1 | 0.984 | 3230 | tags=38%, list=19%, signal=46% |
| EFF_MEM_VS_CENTR_MEM_CD4_TCELL_DN | 139 | 0.35767606 | 1.5071114 | 0.037401576 | 1 | 0.992 | 3085 | tags=31%, list=18%, signal=37% |
| NAIVE_VS_PD1HIGH_CD8_TCELL_UP | 145 | 0.4462093 | 1.6411984 | 0.041753653 | 1 | 0.872 | 3960 | tags=44%, list=23%, signal=57% |
| DC_VS_MAC_DN | 181 | -0.41195267 | -1.7081608 | 0.002 | 1 | 0.736 | 3119 | tags=37%, list=18%, signal=45% |
| LPS_VS_LPS_AND_IL10_STIM_MACROPHAGE_45MIN_DN | 171 | -0.39027083 | -1.6684004 | 0.004016064 | 1 | 0.814 | 4649 | tags=42%, list=27%, signal=57% |
| LOW_DOSE_B_MALAYI_VS_M_TUBERCULOSIS_MAC_UP | 185 | -0.3990984 | -1.6971904 | 0.004048583 | 1 | 0.759 | 3054 | tags=35%, list=18%, signal=42% |
| LAIV_VS_TIV_FLU_VACCINE_DAY7_MONOCYTE_DN | 174 | -0.4134336 | -1.701075 | 0.004166667 | 1 | 0.749 | 2788 | tags=30%, list=16%, signal=36% |
| CTRL_VS_M_TUBERCULOSIS_MAC_UP | 178 | -0.41502163 | -1.7035595 | 0.007905139 | 1 | 0.744 | 3075 | tags=38%, list=18%, signal=46% |
| B2_VS_B1_BCELL_DN | 173 | -0.40560198 | -1.6720842 | 0.008064516 | 1 | 0.81 | 2582 | tags=34%, list=15%, signal=39% |
| CTRL_VS_L_MAJOR_DC_UP | 186 | -0.3616745 | -1.56668 | 0.009689922 | 1 | 0.961 | 3220 | tags=34%, list=19%, signal=41% |
| WT_VS_STAT4_KO_CD8_TCELL_WITH_IFNA_STIM_90MIN_DN | 180 | -0.41964212 | -1.6825293 | 0.012048192 | 1 | 0.793 | 2399 | tags=33%, list=14%, signal=38% |
| CTRL_VS_L_DONOVANI_MAC_UP | 183 | -0.48612732 | -1.731819 | 0.012121212 | 1 | 0.675 | 2848 | tags=46%, list=17%, signal=55% |
| WT_VS_KLF3_KO_BCELL_DN | 181 | -0.3506014 | -1.5241829 | 0.013779528 | 1 | 0.985 | 4683 | tags=39%, list=28%, signal=54% |
| UNSTIM_VS_CPG_STIM_BCELL_UP | 131 | -0.3752568 | -1.559954 | 0.014028057 | 1 | 0.968 | 4338 | tags=42%, list=26%, signal=56% |
| NAIVE_VS_IGM_MEMORY_BCELL_DN | 175 | -0.4197898 | -1.639077 | 0.014084507 | 1 | 0.874 | 3218 | tags=40%, list=19%, signal=49% |
| CTRL_VS_16H_IFNG_IN_CD8POS_DC_DN | 173 | -0.4766018 | -1.7482795 | 0.01446281 | 1 | 0.636 | 2333 | tags=38%, list=14%, signal=43% |
| FOLLICULAR_BCELL_VS_GERMINAL_CENTER_BCELL_DAY40_DN | 178 | -0.37006757 | -1.5691731 | 0.014799154 | 1 | 0.957 | 2734 | tags=30%, list=16%, signal=36% |
| GW1929_VS_PIOGLITAZONE_TREATED_CD4_TCELL_PPARG1_FOXP3_TRANSDUCED_UP | 177 | -0.36207658 | -1.5426247 | 0.016632017 | 1 | 0.977 | 4518 | tags=41%, list=27%, signal=55% |
| UNTREATED_VS_A2AR_AGONIST_TREATED_TREG_DN | 180 | -0.3880413 | -1.5968337 | 0.01775148 | 1 | 0.922 | 3093 | tags=37%, list=18%, signal=44% |
| CD62L_POS_CD56_DIM_VS_CD62L_NEG_CD56_DIM_NK_CELL_DN | 182 | -0.38769066 | -1.6138127 | 0.019011406 | 1 | 0.908 | 4703 | tags=43%, list=28%, signal=59% |
| UNTREATED_VS_TGFB1_TREATED_COMMON_DC_PROGENITOR_UP | 180 | -0.3528345 | -1.5373478 | 0.019027485 | 1 | 0.978 | 3635 | tags=34%, list=21%, signal=43% |
| DC_VS_MAC_M_TUBERCULOSIS_UP | 182 | -0.34777743 | -1.5492659 | 0.019379845 | 1 | 0.975 | 4226 | tags=38%, list=25%, signal=51% |
| BCL6_HET_VS_BCL6_KO_FOLLICULAR_BCELL_DN | 183 | -0.35237408 | -1.574612 | 0.023904383 | 1 | 0.953 | 4607 | tags=42%, list=27%, signal=56% |
| SPLEEN_MACROPHAGE_VS_COLON_TUMORAL_MACROPHAGE_UP | 178 | -0.3575523 | -1.5301718 | 0.024242423 | 1 | 0.98 | 3943 | tags=40%, list=23%, signal=51% |
| UNSTIM_VS_RETINOIC_ACID_STIM_FOLLICULAR_DC_UP | 184 | -0.45734733 | -1.646054 | 0.02631579 | 1 | 0.857 | 3864 | tags=48%, list=23%, signal=62% |
| UNTREATED_VS_IFNA_STIM_STAT4_KO_EFFECTOR_CD8_TCELL_90MIN_UP | 179 | -0.43558338 | -1.6288029 | 0.02631579 | 1 | 0.888 | 4081 | tags=44%, list=24%, signal=57% |
| 4H_VS_16H_IFNG_IN_CD8POS_DC_DN | 181 | -0.44589537 | -1.6570096 | 0.02811245 | 1 | 0.833 | 3678 | tags=42%, list=22%, signal=53% |
| DC_VS_NEUTROPHIL_LPS_STIM_DN | 179 | -0.36961582 | -1.5346032 | 0.03076923 | 1 | 0.979 | 2754 | tags=28%, list=16%, signal=34% |
| CD4_TCELL_VS_B2_BCELL_DN | 175 | -0.37292242 | -1.530472 | 0.030864198 | 1 | 0.98 | 3933 | tags=41%, list=23%, signal=52% |
| RSV_VS_FLU_INF_INFANT_PBMC_DN | 171 | -0.4715193 | -1.6695026 | 0.031185031 | 1 | 0.812 | 2573 | tags=40%, list=15%, signal=47% |
| UNTREATED_VS_PIOGLIZATONE_TREATED_CD4_TCELL_PPARG1_AND_FOXP3_TRASDUCED_DN | 186 | -0.44119453 | -1.639741 | 0.031809144 | 1 | 0.872 | 1977 | tags=32%, list=12%, signal=36% |
| HEALTHY_VS_STAPH_AUREUS_INF_PBMC_DN | 150 | -0.43701473 | -1.5949273 | 0.03373016 | 1 | 0.924 | 3801 | tags=37%, list=22%, signal=47% |
| 0.5H_VS_8H_POLYIC_BMDC_UP | 180 | -0.4010342 | -1.5521368 | 0.033932135 | 1 | 0.972 | 3192 | tags=38%, list=19%, signal=46% |
| CD4_TCELL_VS_B1_BCELL_DN | 179 | -0.41809192 | -1.6224431 | 0.034883723 | 1 | 0.897 | 2788 | tags=34%, list=16%, signal=40% |
| 1H_VS_60H_ACT_CD4_TCELL_WITH_TGFB_IL6_UP | 183 | -0.39473554 | -1.5600793 | 0.034883723 | 1 | 0.968 | 3890 | tags=40%, list=23%, signal=52% |
| WT_VS_MYD88_KO_MACROPHAGE_UP | 185 | -0.4722672 | -1.664783 | 0.034951456 | 1 | 0.822 | 3371 | tags=42%, list=20%, signal=52% |
| EFFECTOR_VS_EXHAUSTED_CD8_TCELL_UP | 176 | -0.45031506 | -1.6241616 | 0.037037037 | 1 | 0.895 | 3666 | tags=45%, list=22%, signal=57% |
| HOFT_CD4_POSITIVE_ALPHA_BETA_MEMORY_T_CELL_BCG_VACCINE_AGE_18_45YO_56D_TOP_100_DEG_AFTER_IN_VITRO_RE_STIMULATION_DN | 42 | -0.49057567 | -1.5717854 | 0.037109375 | 1 | 0.956 | 6424 | tags=79%, list=38%, signal=126% |
| LI_PBMC_MENOMUNE_A_C_Y_W_135_AGE_18_45YO_CORRELATED_WITH_ANTIBODY_RESPONSE_3DY_POSITIVE | 43 | -0.56552655 | -1.6527311 | 0.037523452 | 1 | 0.844 | 4067 | tags=58%, list=24%, signal=76% |
| BLIMP1_VS_LMP1_TRANSDUCED_GC_BCELL_UP | 184 | -0.42304602 | -1.6006904 | 0.041749503 | 1 | 0.919 | 4325 | tags=47%, list=25%, signal=63% |
| DC_VS_MAC_T_GONDII_DN | 187 | -0.3828789 | -1.5695448 | 0.044315994 | 1 | 0.957 | 4311 | tags=41%, list=25%, signal=54% |
| 6H_VS_24H_IL21_TREATED_TCELL_DN | 172 | -0.3951424 | -1.5390327 | 0.04536489 | 1 | 0.977 | 3807 | tags=39%, list=22%, signal=50% |
| CTRL_VS_M_TUBERCULOSIS_DC_DN | 186 | -0.37849844 | -1.5301492 | 0.046875 | 1 | 0.98 | 4280 | tags=41%, list=25%, signal=54% |
| MATSUMIYA_PBMC_MODIFIED_VACCINIA_ANKARA_VACCINE_AGE_18_55YO_VACCINATED_VS_CONTROL_TREATED_IN_VITRO_WITH_MVA85A_6HR_UP | 78 | -0.5264898 | -1.6419324 | 0.047808766 | 1 | 0.869 | 4664 | tags=59%, list=27%, signal=81% |

Supplementary Table 6. The spearman correlation between six diagnostic markers and differential immune cells between INRs and IRs.

| gene | immune_cells | cor | p.value |
| --- | --- | --- | --- |
| FAM120AOS | Activated dendritic cell | -0.304247104 | 0.071579767 |
| FAM120AOS | CD56dim natural killer cell | -0.532818533 | 0.000975789 |
| FAM120AOS | Effector memeory CD8 T cell | -0.373487773 | 0.025499948 |
| FAM120AOS | Immature B cell | -0.362934363 | 0.030233839 |
| FAM120AOS | Natural killer T cell | -0.526383526 | 0.001146774 |
| FAM120AOS | Plasmacytoid dendritic cell | -0.248391248 | 0.143791164 |
| FAM120AOS | T follicular helper cell | -0.705534106 | 3.38E-06 |
| LTA | Activated dendritic cell | -0.27953668 | 0.098824734 |
| LTA | CD56dim natural killer cell | -0.335649936 | 0.045928764 |
| LTA | Effector memory CD8 T cell | -0.34002574 | 0.043039882 |
| LTA | Immature B cell | -0.34980695 | 0.037116001 |
| LTA | Natural killer T cell | -0.298841699 | 0.076962677 |
| LTA | Plasmacytoid dendritic cell | -0.293178893 | 0.082938344 |
| LTA | T follicular helper cell | -0.55984556 | 0.000478829 |
| FAM179B | Activated dendritic cell | -0.422136422 | 0.010887055 |
| FAM179B | CD56dim natural killer cell | -0.560617761 | 0.000468794 |
| FAM179B | Effector memeory CD8 T cell | -0.413384813 | 0.012794872 |
| FAM179B | Immature B cell | -0.367310167 | 0.028189484 |
| FAM179B | Natural killer T cell | -0.527413127 | 0.00111775 |
| FAM179B | Plasmacytoid dendritic cell | -0.415186615 | 0.012380362 |
| FAM179B | T follicular helper cell | -0.625740026 | 6.49E-05 |
| JUN | Activated dendritic cell | -0.226254826 | 0.183932722 |
| JUN | CD56dim natural killer cell | -0.566280566 | 0.000400757 |
| JUN | Effector memeory CD8 T cell | -0.233719434 | 0.169589519 |
| JUN | Immature B cell | -0.076190476 | 0.657684275 |
| JUN | Natural killer T cell | -0.337194337 | 0.044891685 |
| JUN | Plasmacytoid dendritic cell | -0.104761905 | 0.541764255 |
| JUN | T follicular helper cell | -0.427027027 | 0.009931061 |
| PTMA | Activated dendritic cell | -0.287516088 | 0.089270967 |
| PTMA | CD56dim natural killer cell | -0.348005148 | 0.038153825 |
| PTMA | Effector memeory CD8 T cell | -0.272844273 | 0.107429401 |
| PTMA | Immature B cell | -0.466924067 | 0.004475979 |
| PTMA | Natural killer T cell | -0.406692407 | 0.01443961 |
| PTMA | Plasmacytoid dendritic cell | -0.316602317 | 0.060392669 |
| PTMA | T follicular helper cell | -0.682882883 | 8.14E-06 |
| SH3YL1 | Activated dendritic cell | -0.419047619 | 0.011530545 |
| SH3YL1 | CD56dim natural killer cell | -0.531788932 | 0.001001526 |
| SH3YL1 | Effector memory CD8 T cell | -0.292149292 | 0.084062803 |
| SH3YL1 | Immature B cell | -0.275418275 | 0.104054554 |
| SH3YL1 | Natural killer T cell | -0.609266409 | 0.000111191 |
| SH3YL1 | Plasmacytoid dendritic cell | -0.285199485 | 0.091967436 |
| SH3YL1 | T follicular helper cell | -0.524839125 | 0.001191561 |

Supplementary Table 7. miRNA‑mRNA pairs

| **mRNA** | **miRNA** |
| --- | --- |
| FAM120AOS | hsa-miR-3152-3p |
| FAM120AOS | hsa-miR-654-3p |
| FAM120AOS | hsa-miR-3145-3p |
| FAM120AOS | hsa-miR-5684 |
| FAM120AOS | hsa-miR-4714-5p |
| FAM120AOS | hsa-miR-151a-3p |
| FAM120AOS | hsa-miR-4796-5p |
| FAM120AOS | hsa-miR-4766-5p |
| FAM179B | hsa-miR-4666a-5p |
| FAM179B | hsa-miR-3187-5p |
| FAM179B | hsa-miR-4282 |
| FAM179B | hsa-miR-501-5p |
| FAM179B | hsa-miR-5692a |
| FAM179B | hsa-miR-30a-5p |
| FAM179B | hsa-miR-4719 |
| FAM179B | hsa-miR-7-1-3p |
| FAM179B | hsa-miR-30b-5p |
| FAM179B | hsa-miR-513c-3p |
| FAM179B | hsa-miR-3143 |
| FAM179B | hsa-miR-557 |
| FAM179B | hsa-miR-30c-5p |
| FAM179B | hsa-miR-3935 |
| FAM179B | hsa-miR-101-3p |
| FAM179B | hsa-miR-1285-3p |
| FAM179B | hsa-miR-30d-5p |
| FAM179B | hsa-miR-32-3p |
| FAM179B | hsa-miR-4307 |
| FAM179B | hsa-miR-454-3p |
| FAM179B | hsa-miR-4262 |
| FAM179B | hsa-miR-30e-5p |
| FAM179B | hsa-miR-4495 |
| FAM179B | hsa-miR-3974 |
| FAM179B | hsa-miR-3163 |
| FAM179B | hsa-miR-7-2-3p |
| FAM179B | hsa-miR-181a-5p |
| FAM179B | hsa-miR-4501 |
| FAM179B | hsa-miR-181b-5p |
| FAM179B | hsa-miR-4452 |
| FAM179B | hsa-miR-4803 |
| FAM179B | hsa-miR-580-3p |
| FAM179B | hsa-miR-181c-5p |
| FAM179B | hsa-miR-3591-5p |
| FAM179B | hsa-miR-513a-3p |
| FAM179B | hsa-miR-181d-5p |
| JUN | hsa-miR-513c-5p |
| JUN | hsa-miR-3925-5p |
| JUN | hsa-miR-429 |
| JUN | hsa-miR-580-3p |
| JUN | hsa-miR-200b-3p |
| JUN | hsa-miR-200c-3p |
| JUN | hsa-miR-203a |
| JUN | hsa-miR-4311 |
| JUN | hsa-miR-4729 |
| JUN | hsa-miR-633 |
| JUN | hsa-miR-4504 |
| JUN | hsa-miR-514b-5p |
| JUN | hsa-miR-5011-5p |
| JUN | hsa-miR-15a-3p |
| JUN | hsa-miR-1299 |
| JUN | hsa-miR-4529-5p |
| JUN | hsa-miR-4533 |
| JUN | hsa-miR-495-3p |
| JUN | hsa-miR-1277-5p |
| JUN | hsa-miR-4693-3p |
| LTA | hsa-miR-593-3p |
| LTA | hsa-miR-4775 |
| LTA | hsa-miR-24-1-5p |
| LTA | hsa-miR-24-2-5p |
| PTMA | hsa-miR-3120-3p |
| PTMA | hsa-miR-548p |
| PTMA | hsa-miR-3658 |
| PTMA | hsa-miR-548av-5p |
| PTMA | hsa-miR-7-2-3p |
| PTMA | hsa-miR-1972 |
| PTMA | hsa-miR-3613-3p |
| PTMA | hsa-miR-624-3p |
| PTMA | hsa-miR-3163 |
| PTMA | hsa-miR-377-3p |
| PTMA | hsa-miR-512-5p |
| PTMA | hsa-miR-3926 |
| PTMA | hsa-miR-4495 |
| PTMA | hsa-miR-513a-3p |
| PTMA | hsa-miR-548k |
| PTMA | hsa-miR-513c-3p |
| PTMA | hsa-miR-7-1-3p |
| PTMA | hsa-miR-129-5p |
| PTMA | hsa-miR-545-3p |
| PTMA | hsa-miR-3918 |
| PTMA | hsa-miR-367-5p |
| PTMA | hsa-miR-659-3p |
| PTMA | hsa-miR-3148 |
| SH3YL1 | hsa-miR-23a-3p |
| SH3YL1 | hsa-miR-23b-3p |
| SH3YL1 | hsa-miR-1303 |
| SH3YL1 | hsa-miR-23c |
| SH3YL1 | hsa-miR-595 |
| SH3YL1 | hsa-miR-548l |
| SH3YL1 | hsa-miR-4282 |
| SH3YL1 | hsa-miR-590-3p |
| SH3YL1 | hsa-miR-4775 |
| SH3YL1 | hsa-miR-4804-3p |
| SH3YL1 | hsa-miR-4777-3p |
| SH3YL1 | hsa-miR-3973 |
| SH3YL1 | hsa-miR-4521 |
| SH3YL1 | hsa-miR-4474-3p |
| SH3YL1 | hsa-miR-4697-3p |

Supplementary Table 8. mRNA TF pairs.

| **mRNA** | **TF** |
| --- | --- |
| PTMA | NFIC |
| PTMA | ESR1 |
| PTMA | RELA |
| PTMA | E2F1 |
| PTMA | SP1 |
| PTMA | TFAP2A |
| PTMA | E2F6 |
| PTMA | HINFP |
| PTMA | ZNF354C |
| JUN | E2F6 |
| JUN | TFAP2A |
| JUN | SP1 |
| JUN | SREBF1 |
| JUN | FOXC1 |
| JUN | FAM120AOS |
| JUN | BRCA1 |
| FAM120AOS | HNF4A |
| FAM121AOS | FOXL1 |
| FAM122AOS | TP53 |
| FAM123AOS | FOXC1 |
| LTA | FOXL1 |
| LTA | SRF |
| LTA | TEAD1 |
| LTA | SREBF1 |
| LTA | FOXC1 |
| SH3YL1 | TP53 |
| SH3YL1 | SP1 |
| SH3YL1 | E2F1 |
| SH3YL1 | USF2 |
| SH3YL1 | PRRX2 |
| SH3YL1 | RUNX2 |
| SH3YL1 | HOXA5 |
| SH3YL1 | NFKB1 |
| SH3YL1 | MAX |
| SH3YL1 | NRF1 |
| SH3YL1 | GATA2 |
| SH3YL1 | SREBF1 |
| SH3YL1 | FOXC1 |
